# Rehabilitation in primary care for an ageing population: a secondary analysis from a scoping review of rehabilitation delivery models

**DOI:** 10.1186/s12913-023-10387-w

**Published:** 2024-01-23

**Authors:** Vanessa Seijas, Roxanne Maritz, Satish Mishra, Renaldo M Bernard, Patricia Fernandes, Viola Lorenz, Barbara Machado, Ana María Posada, Luz Helena Lugo-Agudelo, Jerome Bickenbach, Carla Sabariego

**Affiliations:** 1 Faculty of Health Sciences and Medicine, University of Lucerne, Alpenquai 4, Lucerne, 6005 Switzerland; 2 Center for Rehabilitation in Global Health Systems (WHO Collaborating Center), Faculty of Health Sciences and Medicine, University of Lucerne, Lucerne, Switzerland; 3 Ageing, Functioning Epidemiology and Implementation, Swiss Paraplegic Research, Nottwil, Switzerland; 4 Disability, Rehabilitation, Palliative and Long-Term Care, Health Workforce and Service Delivery Unit, Division of Country Health Policies and Systems, WHO Regional Office for Europe, UN City, Marmorvej 51, Copenhagen, 2100 Denmark; 5 Department of Clinical Medicine, Federal University of Parana, R. XV de Novembro, 1299 - Centro, Curitiba, PR 80060-000 Brasil; 6 Rehabilitation in Health Research Group, Sede de Investigación Universitaria, University of Antioquia, Cl. 62 # 52-59, Medellín, Colombia

**Keywords:** Healthy ageing, Older persons, Primary health care, Rehabilitation, Health services delivery, And health policy

## Abstract

**Background:**

The world population is ageing rapidly. Rehabilitation is one of the most effective health strategies for improving the health and functioning of older persons. An understanding of the current provision of rehabilitation services in primary care (PC) is needed to optimise access to rehabilitation for an ageing population. The objectives of this scoping review are a) to describe how rehabilitation services are currently offered in PC to older persons, and b) to explore age-related differences in the type of rehabilitation services provided.

**Methods:**

We conducted a secondary analysis of a scoping review examining rehabilitation models for older persons, with a focus on PC. Medline and Embase (2015–2022) were searched to identify studies published in English on rehabilitation services for people aged 50 + . Two authors independently screened records and extracted data using the World Health Organization (WHO)’s operational framework, the Primary Health Care Systems (PRIMASYS) approach and the WHO paper on rehabilitation in PC. Data synthesis included quantitative and qualitative analysis.

**Results:**

We synthesised data from 96 studies, 88.6% conducted in high-income countries (HICs), with 31,956 participants and identified five models for delivering rehabilitation to older persons in PC: community, home, telerehabilitation, outpatient and eldercare. Nurses, physiotherapists, and occupational therapists were the most common providers, with task-shifting reported in 15.6% of studies. The most common interventions were assessment of functioning, rehabilitation coordination, therapeutic exercise, psychological interventions, and self-management education. Environmental adaptations and assistive technology were rarely reported.

**Conclusions:**

We described how rehabilitation services are currently provided in PC and explored age-related differences in the type of rehabilitation services received. PC can play a key role in assessing functioning and coordinating the rehabilitation process and is also well-placed to deliver rehabilitation interventions. By understanding models of rehabilitation service delivery in PC, stakeholders can work towards developing more comprehensive and accessible services that meet the diverse needs of an ageing population. Our findings, which highlight the role of rehabilitation in healthy ageing, are a valuable resource for informing policy, practice and future research in the context of the United Nations Decade of Healthy Ageing, the Rehab2030 initiative and the recently adopted WHA resolution on strengthening rehabilitation in health systems, but the conclusions can only be applied to HICs and more studies are needed that reflect the reality in low- and middle-income countries.

**Supplementary Information:**

The online version contains supplementary material available at 10.1186/s12913-023-10387-w.

## Background

The global population is undergoing a significant demographic transition characterised by an increasing proportion of older persons. By 2050, approximately 2.1 billion people aged 60 and older will constitute over 22% of the global population, with 80% residing in low- and middle-income countries (LMICs) [1]. This transition presents unprecedented challenges for societies and healthcare systems as they strive to meet the evolving needs of an ageing population and address exacerbated disparities in healthcare access and the ability to meet basic needs in older age [2]. In response, the World Health Assembly (WHA) and the United Nations (UN) General Assembly declared 2021–2030 as the Decade of Healthy Ageing (hereafter referred to as “The Decade”) [3]. Healthy ageing is defined by the World Health Organization (WHO) as “*the process of developing and maintaining the functional ability that enables well-being in older age*”, while functional ability results from the interaction between a person’s intrinsic capacity and their environment [2]. Consequently, the physical, social, attitudinal, and political environments – including access to health care that meets the needs of an ageing population – are crucial determinants of differences in functional ability among older persons across countries.

A country’s healthcare system, including strong primary health care (PHC), “*a whole-of-society approach to health that aims to maximise the level and distribution of health and well-being*” [4], is a key environmental factor influencing people’s functional ability. The performance of healthcare systems has been associated with important community health outcomes [5]. In particular, the availability of a strong PHC system leads to enhanced access to healthcare, better health outcomes and more health equity [6–8], which is why strengthening PHC has been considered by countries signing the Astana declaration [9] as the most inclusive, effective, and efficient approach to improve the health and well-being of the population. PHC is a cornerstone of sustainable health systems with universal health coverage (UHC) [9]. In line with this, *The decade* has defined as a priority area for action “*deliver person-centred integrated care and primary health services that are responsive to older people*” [2] and has produced guidelines on how to improve the functional ability of older persons in PHC [10–12]. Rehabilitation is one of the five essential health strategies mentioned in the Astana declaration that is suitable to be provided at PHC [13].

Although rehabilitation is a key person-centred health strategy for achieving the goals of the Decade, health systems worldwide are generally not meeting the rehabilitation needs of older persons. Rehabilitation, defined by the WHO as “*a set of interventions designed to optimize functioning and reduce disability in persons with health conditions in interaction with their environment” *[14], is one of the key health services to improve the functional ability of an ageing population and is therefore instrumental to moving the Astana declaration forward [15]. Rehabilitation needs in an ageing population are driven by the increased prevalence of non-communicable diseases and multimorbidity [16]. Globally, in 2019, an estimated 2.4 billion people had conditions that would have benefited from rehabilitation. These rehabilitation needs have increased by 63% since 1900 mainly due to population growth and ageing [17]. However, most of these needs are still unmet. In LMICs, as many as 50% of people do not receive the rehabilitation services they need [18].

While prevention is essential to avoid or delay the occurrence of chronic diseases, rehabilitation is key to optimising a person’s functioning considering already existing chronic health conditions and multimorbidity [19]. Specifically, rehabilitation fully takes into account the physical, social, political and economic environment of a person [15]. Indeed, a review aiming at identifying evidence gaps in health, social care and technological interventions to improve the functional ability of older persons showed that the most commonly used intervention was home-based rehabilitation [20]. Another review found that education, skills training, exercise, and physical activity—traditional rehabilitation interventions—were the most commonly provided interventions to improve the functional ability of older persons in rural and remote areas [21]. Recognising the potential of rehabilitation, the WHA adopted in May of 2023 a resolution for “*Strengthening rehabilitation in health systems”,* stating that the provision of expanding rehabilitation to all levels of health, from primary to tertiary is essential to ensure the availability and affordability of quality and timely rehabilitation services for all [22].

Policymakers responsible for strengthening rehabilitation in health systems for ageing populations could benefit from a sound knowledge of how rehabilitation services are currently delivered in primary care (PC)—*a component of PHC and a key process in the health system that supports first-contact, accessible, continuous, comprehensive and coordinated patient-centred care*” [4]. In 2009, a scoping review identified and characterised models for integrating rehabilitation into PC but did not focus on the specific needs of older persons [23]. To address this gap, we conducted a scoping review to identify models of rehabilitation service delivery for older persons [24]. We used the term “model” to describe the approach used to deliver rehabilitation services to the person. Six models were identified: Outpatient rehabilitation: Patients receive rehabilitation in a healthcare facility and return home after the day’s session(s). Inpatient rehabilitation: Rehabilitation is provided during inpatient episodes of care. Home rehabilitation: Rehabilitation is carried out in the patient’s home through visits from rehabilitation workers. Telerehabilitation: Rehabilitation is delivered using communication technologies (e.g. computers, telephones or smartphones). Rehabilitation in the community: Rehabilitation is provided in a community setting, such as a community centre or recreational area. Eldercare rehabilitation: Rehabilitation is provided in eldercare facilities such as assisted living, adult day care, long-term care, nursing homes (often called residential care), hospice care, and home care.

The studies included in this scoping review were further classified into three categories according to the level of care: PC, specialised care – including secondary or tertiary level– and a combination of the two levels, for example, services starting in a university hospital but continuing with a community-based exercise programme. However, due to the large amount of information, we were not able to do a detailed description of the rehabilitation services in PC. We, therefore, decided to conduct a secondary analysis of the above-mentioned scoping review with the following objectives: a) to describe how rehabilitation services are currently offered in PC to older persons by rehabilitation service delivery models, and b) to explore age-related differences in the type of rehabilitation services provided. Our findings can contribute to the (re)design of rehabilitation services in PC to meet the growing needs of an ageing population.

## Methods

### Study design

We conducted a secondary analysis of a scoping review [24] that aimed to examine rehabilitation service delivery models for optimising the intrinsic capacity and functional ability of older persons. We defined 2 new research questions for this secondary analysis using the framework PCC (Population, Concept, Context): a) How are rehabilitation services currently offered in PC to people over 50 years of age, in terms of service delivery models, providers and rehabilitation interventions? b) Are there age-related differences in rehabilitation services provided in PC to people over 50 years of age? A scoping review was considered the best method to address these questions as it is particularly useful for mapping the available evidence and providing a broad overview of the existing literature, identifying gaps in knowledge, clarifying concepts and informing the design of systematic reviews in emerging areas of study, such as the integration of rehabilitation into PC [25, 26]. We followed state-of-the-art methods [27] to conduct this review and the Preferred Reporting Items for Systematic Reviews and Meta-Analyses extension for Scoping Reviews (PRISMA-ScR) [28] to guide its reporting (checklist available as Additional file 3). The protocol of the primary review and the protocol addendum for this secondary analysis are available as Additional files 1 and 2.

### Eligibility criteria

Key selection criteria of the primary scoping review included participants’ mean age above 50 and a focus on rehabilitation provision models rather than individual interventions; the full criteria are available here [24]. We used a mean age above 50 based on evidence showing that countries with similar levels of age-related burden experience different onsets of ageing, with the lowest starting around 50 years [29]. This secondary analysis was restricted to papers classified as PC. PHC is defined by the WHO as “a whole-of-society approach *to health that aims to maximise the level and distribution of health and well-being through three components: (a) primary care and essential public health functions as the core of integrated health services; (b) multisectoral policies and actions; and (c) empowered people and communities*” [4]. Primary care (PC) is further defined as “*a key process in the health system that supports first-contact, accessible, continuous, comprehensive and coordinated patient-centred care*” [4]. In this review, we focused on PC. We included studies if: a) the paper self-identified as PC or PHC, or b) the rehabilitation interventions were delivered exclusively by PC workers (e.g., nurses or general practitioners (or “family doctors”)) in a traditional PC setting (e.g., home or community), and/or the interventions delivered did not require complex equipment or highly specialised training. By general practitioners (or “family doctors”) we refer to those medical doctors who “*assume responsibility for the provision of continuing and comprehensive medical care to individuals, families and communities. Including general practitioners—District medical doctors—therapists—Family medical practitioners (“family doctors”)—Medical interns or residents specialising in general practice*” [30].

### Information sources and search strategy

Given rehabilitation’s broad scope [31] and varying interpretations across countries and settings [32], a comprehensive search strategy was developed in the primary scoping review aiming to capture a wide range of rehabilitation models [24]. A comprehensive search of Medline and Embase was conducted from 2015, the year in which the World Report on Ageing and Health (WRAH) [33] was published, to May 2022, covering the concepts of ageing, older persons, rehabilitation, and health services. The search was complemented by scanning reference lists of systematic reviews identified during the first eligibility check. The search concepts, terms, and full electronic search strategies for each bibliographic database queried are available in Additional file 4.

### Study selection process

Expecting a high number of records due to the comprehensive search strategy, a random 35% sample of titles and abstracts was screened independently by two authors in the primary scoping review [24], using Rayyan software [34]. Training rounds continued until over 90% agreement was reached, with conflicts resolved by a third author. For this secondary analysis, we excluded papers that were classified as being provided at specialised levels of care or at a combination of levels, PC and specialised levels of care.

### Data extraction process

Data on study characteristics, target populations, and rehabilitation service characteristics, including setting, level of care, professionals, interventions, and dosage, were extracted in the primary review [24]. For the secondary analysis, we complemented these data with specific PC data using the WHO’s operational framework for primary health care [13], the PRIMASYS approach [35], and the WHO background paper on integrating rehabilitation in PC (World Health Organisation, Rehabilitation Programme, Sensory Functions, Disability, and Rehabilitation Unit, Department of Family and Community Medicine at the University of Toronto: Integrating rehabilitation in primary: background paper, unpublished) as conceptual frameworks. Additional data on study settings, stakeholder involvement in planning rehabilitation services, and the dosage of rehabilitation programs were extracted. Dosage refers to the sum of the number, frequency and duration of each rehabilitation session and the total length of the programme. Two authors (VL and BM) independently extracted additional data after achieving more than 90% agreement in pilot tests, and any discrepancies were resolved with the first author (VS).

We did not appraise methodological quality or risk of bias, in line with scoping reviews’ methodology [25, 27], and with our goal of identifying and describing rehabilitation delivery models rather than assessing whether the interventions or strategies used were effective. Further details are provided in Additional files 1 and 2.

### Data synthesis

New data synthesis included descriptive quantitative analysis (e.g., frequencies) of study characteristics, interventions, and service provision, and qualitative analysis as well as an iterative approach for defining the characteristics of rehabilitation services and emerging topics. In this secondary analysis we kept the same categorization for “models of rehabilitation service delivery”. In the primary review [24], ‘mode of service delivery’, collected during data extraction according to the International Classification of Service Organization In Rehabilitation (ICSO-R 2.0) [36], emerged as natural categories for organising and describing rehabilitation models during data synthesis. The mode of service delivery is defined by the ICSO-R 2.0 as “*The way services are delivered to the users. Inclusions: Inpatients, outpatients, day hospital/day service, home and community, tele-rehabilitation, or any other setting for service delivery*” [36].

We used the International Classification of Health Interventions (ICHI) [37], and the WHO Packages of Interventions for Rehabilitation (PIR) [38] included in the Universal Health Coverage (UHC) Compendium to categorise rehabilitation interventions in the primary review [24]. The term ‘rehabilitation intervention’ corresponds to the ‘action’ level in the ICHI and UHC taxonomies. Considering the WHO [14] definition of rehabilitation, we defined rehabilitation interventions as any action taken by health or allied health professionals to optimise functioning and reduce disability, which requires resources (time, equipment, consumables, knowledge) to be provided. In the primary review [24], we identified seven categories of rehabilitation interventions: assessment, coordination and management of the rehabilitation process, pharmacological agents, restorative and compensatory approaches, provision of assistive technology (AT), environmental adaptations (EAs), and education and advice. In this secondary analysis, we revised the names of the categories considering input from new co-authors with expertise in PC. Further details about the synthesis process can be found in Additional files 1 and 2.

### Patient and public involvement statement

We did not involve older people, patients or patient representatives in the methodological design, conduct, reporting or dissemination plan of the scoping reviews.

## Results

### Study selection and characteristics

**Fig. 1:**
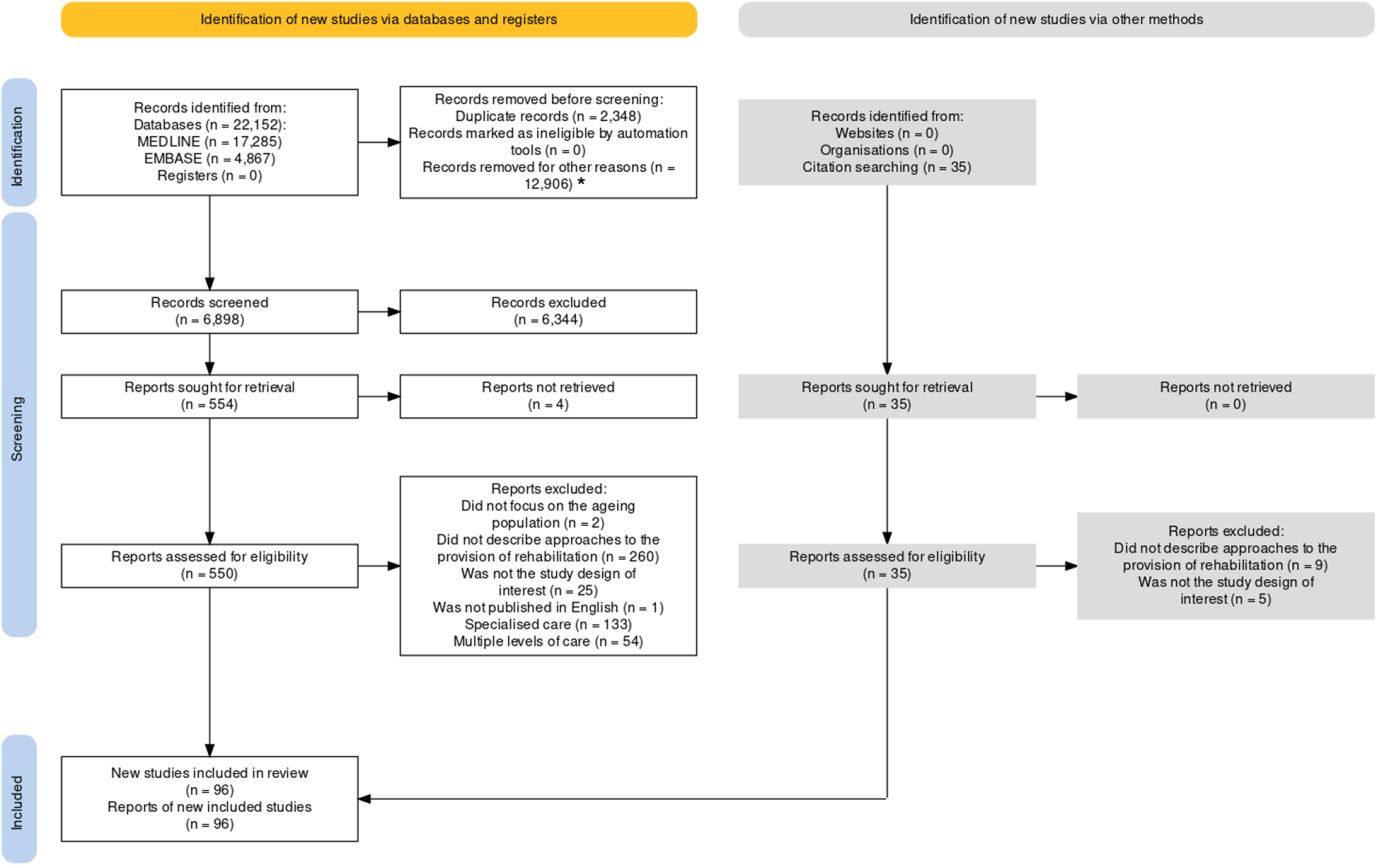
Preferred Reporting Items for Systematic Reviews and Meta-Analyses extension for Scoping Reviews (PRISMA-ScR) [39]. Search of May 31, 2022. *Excluded by random sampling of 65%

In the primary scoping review, 6,898 titles and abstracts were screened, and 283 studies were included [24]. Of these, 96 studies (33.9%), with 31,956 participants, were classified as PC and included in this secondary analysis (Fig. 1 shows the PRISMA flowchart).

Included PC studies were published between 2015 and 2022; 88.6%, were conducted in high-income countries (HICs), mostly in the Netherlands, the United States (US) and the United Kingdom (UK); only one study was conducted in a low-income setting, Uganda [40] (see Table 1). Most studies were interventional 83.3%, with a sample size ranging from 5 [41, 42] to 8,217 [43] participants, and did not involve patients or their representatives in the rehabilitation program design or implementation (83.3%). Half of the studies that reported the setting were conducted in rural or semi-rural areas (18.8%). The main characteristics of all included studies are available in Additional file 5, Tables S1 and S2.

**Table 1:** Characteristics of the included studies

Category	Details	N	% within category	% of included studies
**Year of publication**	2015	14		14.6
	2016	21		21.9
	2017	8		8.3
	2018	19		19.8
	2019	4		4.2
	2020	2		2.1
	2021	23		24.0
	2022	5		5.2
**Country**	The Netherlands	11	11.3	11.5
	United States	11	11.3	11.5
	United Kingdom	10	10.3	10.4
	Australia	8	8.3	8.3
	Korea	6	6.2	6.3
	Spain	6	6.2	6.3
	China	6	6.2	6.3
	Norway	5	5.2	5.2
	Japan	4	4.1	4.2
	Taiwan, China	4	4.1	4.2
	Canada	3	3.1	3.1
	Less than 3 studies^a^	23	23.7	24.0
	Total countries reported	97	100	101.0
**Study design**	Intervention study	80		83.3
	Observational Study	12		12.5
	Qualitative study	4		4.2
**Study codesign**	Yes^b^	12		12.5
	No or not reported	84		87.5
**Setting**	Rural/Semi-rural	18	18	18.8
	Urban	19	19	19.8
	Not reported	63	63	65.6
	Total settings reported	100		
**Most used age-related inclusion criteria**	Older than 65	29		30.2
	Limitation by age	6		6.2
	Older than 18	17		17.7
	Another criterion	22		22.9
	Not reported	22		22.9
**Participants’ sex predominance**	Female predominance	54		56.3
	Balanced	27		28.1
	Male predominance	7		7.3
	Not reported	8		8.3
**Target population**	People with a decline in functioning/functional ability^c^	50		52.1
	People with a single health condition	39		40.6
	People with more than two health conditions	7		7.3
**Health condition area**	Neurological	12	30	12.5
	Cardiovascular	8	20	8.3
	Respiratory	8	20	8.3
	Metabolic	4	10	4.2
	Musculoskeletal	3	7.5	3.1
	Pain	2	5	2.1
	Autoimmune disease	1	2.5	1.0
	Cancer	1	2.5	1.0
	Communicable diseases	1	2.5	1.0
	Number of health conditions reported	40	100	41.7
**Health condition**	Stroke	9	22.5	9.4
	COPD	8	20	8.3
	Cardiovascular disease not specified	3	7.5	3.1
	Diabetes	3	7.5	3.1
	Heart failure	3	7.5	3.1
	Less than 3 studies^d^	14	35	14.6
	Number of health conditions reported	40	100	41.7
**Distribution of participant’s mean age**		**N**	**mean**	**SD**
	Quantile 1	21	57.8	3.7
	Quantile 2	17	67.7	2.4
	Quantile 3	31	74.9	2.3
	Quantile 4	21	81.8	1.8

^a^Countries with less than 4 studies: Austria, Belgium, Brazil, Cyprus, Finland, Greece, Ireland, Mexico, New Zealand, Singapore, Uganda, Denmark, Germany, Italy, Portugal, Sweden, Turkey, Canada

^b^Study participants were involved in the design of the rehabilitation program and research study

^c^We considered “functional ability” and “functioning”, as introduced in the International Classification of Functioning, Disability and Health (ICF) [44] as equivalent concepts

^d^Health conditions with less than 3 studies: Chronic pain, Cognitive Impairment, Osteoarthritis, COVID-19, Cancer not specified, Coronary heart disease, Inflammatory arthritis, Multiple sclerosis, Peripheral artery disease, Rotator cuff tendinopathy

### Beneficiaries of rehabilitation programmes (Table 1)

In 56.3% of the studies, females accounted for more than 60% of the study participants. In 52.1% of the studies, participants’ eligibility was based on functional decline, while 40.6% of the studies were disease-oriented. Of the latest, the most common health condition was stroke, followed by chronic obstructive pulmonary disease (COPD). The most common age-related inclusion criterion was “older than 65 y/o”, and the participants’ mean age across studies was 71.1(SD 9.1).

### Rehabilitation service delivery (Table 2)

**Table 2:** Characteristics of the rehabilitation programs

Category	Details	N	% within category	% of included studies
**Rehabilitation service delivery model**	Community	46	40.7	47.9
	Home	27	23.9	28.1
	Telerehabilitation	19	16.8	19.8
	Outpatient	15	13.3	15.6
	Eldercare	6	5.3	6.3
	Models identified	113	100.0	117.7
**Service provider**	Health workers	83	84.7	86.5
	Peers and volunteers	7	7.1	7.3
	Informal caregivers and family	4	4.1	4.2
	Not reported	4	4.1	4.2
	Providers identified	98	100.0	102.1
**Type of rehabilitation intervention provided**				
** Assessment**		**59**	**23.3**	**61.5**
	Person-centred goals	39	36.8	40.6
	Functioning/functional ability^a^	25	23.6	26.0
	Fall risk	9	8.5	9.4
	Environment	8	7.6	8.3
	Medications used	7	6.6	7.3
	Comprehensive geriatric assessment	5	4.7	5.2
	Health status	5	4.7	5.2
	Emotional functions	4	3.8	4.2
	Less than two studies^b^	4	4	4.2
** Care coordination and management**		**45**	**17.8**	**46.9**
	Follow up visits	25	30.5	26.0
	Case management	15	18.3	15.6
	Monitoring of functioning/functional ability^a^	14	17.1	14.6
	Health status monitoring	11	13.4	11.5
	Rehabilitation process coordination and management	10	12.2	10.4
	Home visit	6	7.3	6.3
	Discharge planning	1	1.2	1.0
** Restorative and compensatory approaches**		**78**	**30.8**	**81.3**
	Therapeutic exercise	52	41.6	54.2
	Motivational interventions	12	9.6	12.5
	Multicomponent care or rehabilitation program not specified	10	8.0	10.4
	Activities of daily living skills training	10	8.0	10.4
	Behavioural interventions	8	6.4	8.3
	Cognitive rehabilitation	6	4.8	6.3
	Psychological interventions not specified	5	4.0	5.2
	Therapeutic recreation	5	4.0	5.2
	Management of incontinence	4	3.2	4.2
	Occupational therapy not specified	4	3.2	4.2
	Less than two studies^c^	9	7.2	9.4
** Education, counselling and skills training**		**60**	**23.7**	**62.5**
	Education and skills training for selfcare and self-management not specified	46	45.1	47.9
	Education and skills training for caregivers	16	15.7	16.7
	Education and counselling on behavioural risk factors	14	13.7	14.6
	Education and counselling on self-directed therapeutic exercise	13	12.8	13.5
	Education and counselling about healthy diet and nutrition	12	11.8	12.5
	Education and counselling for weight management	1	1.0	1.0
** Environmental adaptations**		**7**	**2.8**	**7.3**
** Provision and training in the use of assistive technology**		**4**	**1.6**	**4.2**
**Time and intensity of rehabilitation program**^**d**^				
** Dosage decision**	Adapted	16		16.67
	Prespecified	50		52.08
	Prespecified and adapted	30		31.25
		**median**	**IQR**	**min–max**
** Frequency of sessions per week **		1.0	0.55–2	0.07–8
**Session duration in minutes **		65.0	50–120	10–300
**Number of sessions per patient per program **		16.0	8–29	1–149
**Rehabilitation length in weeks **		12.0	8–28	2–192
**Health workers **	Physical therapists	38	22.0	39.6
	Nurses	34	19.7	35.4
	Occupational Therapists	16	9.3	16.7
	General practitioners (or “family doctors”)	10	5.8	10.4
	Social workers	9	5.2	9.4
	Dieticians	9	5.2	9.4
	Exercise professionals	7	4.1	7.3
	Community workers	6	3.5	6.3
	Other physicians^e^	6	3.5	6.3
	Geriatricians	5	2.9	5.2
	Psychologists	3	1.7	3.1
	Rehabilitation physicians	2	1.2	2.1
	Speech and language therapists	2	1.2	2.1
	Other^f^	11	6.4	11.5
	Does not applied or not reported	15	8.7	15.6
	Health workers identified	173	100.0	180.2

^a^We considered “functional ability” and “functioning”, as introduced in the International Classification of Functioning, Disability and Health (ICF) [44] as equivalent concepts

^b^Other assessments included: Cognitive functions, family and caregivers support network, and nutritional status

^c^Other restorative approaches included: Pharmacological agents not specified, Problem solving skills training, Physical therapy not specified, social skills training, and Music therapy

^d^Medians and IQRs are presented because all dosage-related variables were not normally distributed

^e^Other physicians included cardiologists and physicians not specified

^f^Other health workers included professional caregivers, home care personnel, dance instructors, welfare officers, pharmacists, mental health workers or therapists not specified, health care managers, music therapists, therapist assistants or students, gerontologists, optometrists

We identified five models for delivering rehabilitation services at PC: community, home-based, telerehabilitation, outpatient and eldercare. Almost 50% of the studies utilised the community model, in which services are delivered in a community setting, such as a community centre or public recreational area. The second most used model was home-based rehabilitation (28.1%), which involves providing services at the person’s home as an integrated part of PC outreach services. The telerehabilitation model, another outreach strategy, and the outpatient model, where the person goes to a healthcare facility to receive services and then returns home, were used in 20% and 15.6% of the studies, respectively. The least common model was eldercare-based rehabilitation (6.3%), which involves providing rehabilitation services at eldercare facilities.

**Fig. 2:**
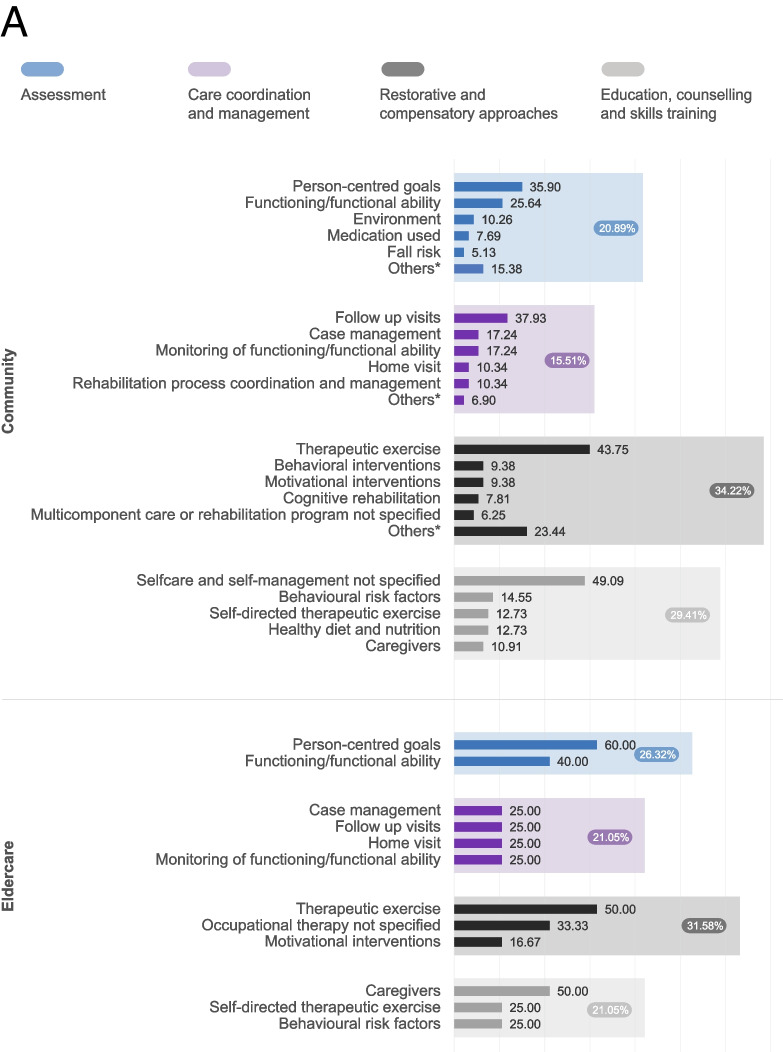

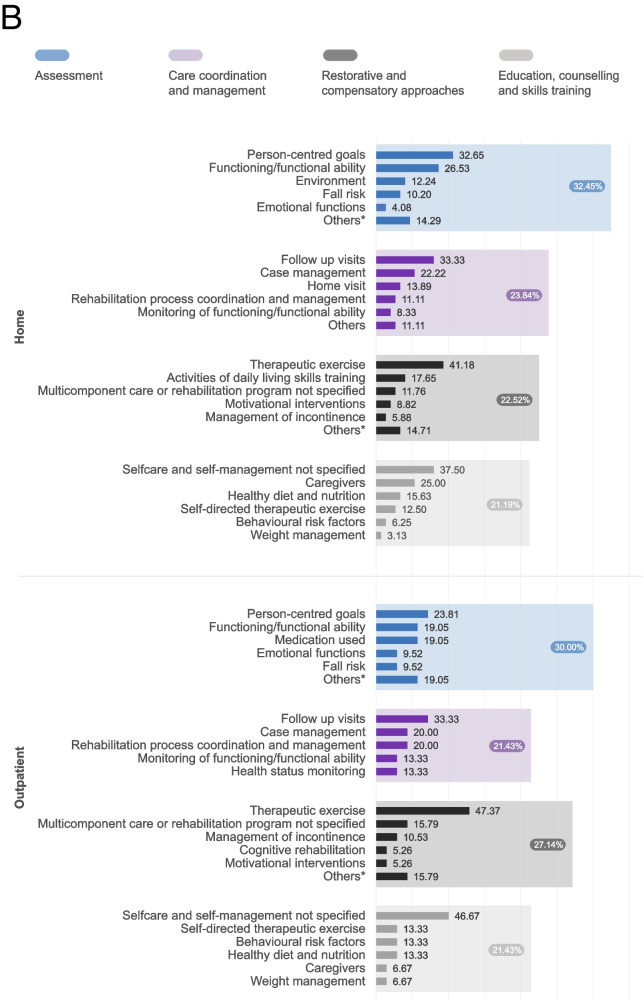

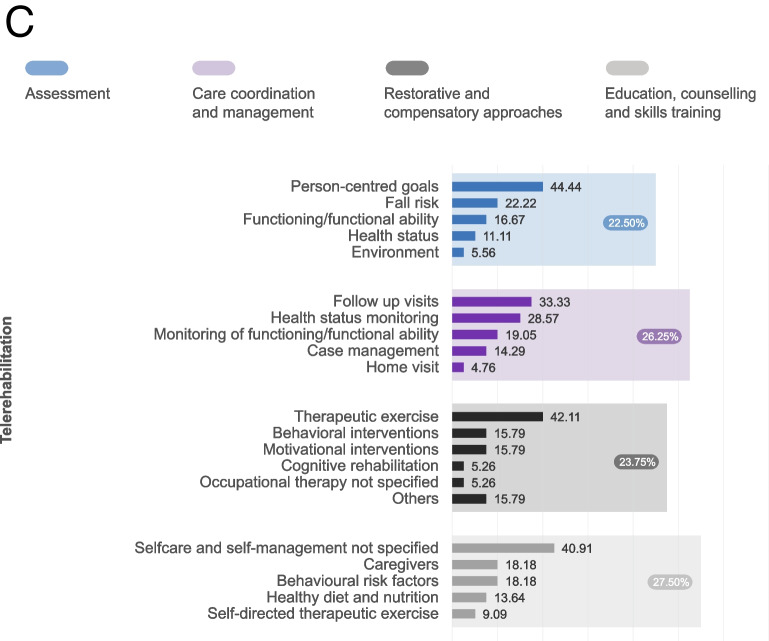
Frequency of rehabilitation interventions by categories and models of rehabilitation service delivery

The types of rehabilitation interventions provided by the five models were grouped into six categories: 1) assessment, 2) care coordination and management, 3) restorative and compensatory approaches, including pharmacological agents (used in 81.3% of studies), 4) education, counselling, and skills training, 5) environmental adaptations, and 6) provision and training in the use of assistive technology. Figure 2 displays the frequency of the 253 identified rehabilitation interventions across the first four categories (the most common ones) by models of rehabilitation delivery. Interventions delivered in the community and eldercare models primarily focused on restorative and compensatory approaches, including interventions such as therapeutic exercises, activities of daily living (ADL) skills training, and cognitive rehabilitation. The home and outpatient models, on the other hand, placed a greater emphasis on assessment, including the evaluation of functioning, emotional functions, and the risk of secondary complications. Finally, the telerehabilitation model primarily focused on education, counselling, and skills training, including interventions such as self-directed therapeutic exercise education, caregiver education and training, and weight management. Psychological interventions were the second most provided interventions across all models: cognitive training, behavioural, and motivational interventions were among the top five interventions in the telerehabilitation and community models and at least one of them was among the top 5 in all models. The dosage of rehabilitation was very heterogeneous between models, but on average patients received 16 sessions, once a week for 12 weeks, and 52.1% of the rehabilitation programmes had a pre-determined dosage that was not adapted to the patient’s needs and progress.

Health workers, mostly physical therapists (PTs), nurses and occupational therapists (OTs), delivered the interventions in 86.5% of the studies. Fifteen studies (15.6%) reported some form of role or task shifting, of which all but two were conducted in HICs: one in China (upper-middle-income country) and one in Uganda (low-income country). The most common types of task shifting were from health workers to either the patient’s caregivers [40, 45, 46] or peers and volunteers [47–50] as well as from specialised rehabilitation workers to primary care workers [43, 51]. Twenty-four studies (25%) reported multidisciplinary teams (three different health workers or more) working at PC, mostly including nurses, geriatricians, and general practitioners (or “family doctors”) [52–54]; or PTs, OTs and other types of physicians [46, 55–57]. The main characteristics of the rehabilitation programmes and dosage information of each included study are available as Additional files 5 and 6, Tables S3 and S4.

### Rehabilitation characteristics by age groups (Table 3)

**Table 3:** Rehabilitation program characteristics by age groups of participants mean age

Category	Details (min/max)	Group 50 to 60^a^ (50.2/63.3)	Group 60 to 70^b^ (63.4/71.6)	Group 70 to 80^c^ (71.7/78.6)	Group older than 80^d^ (79/87.3)
		**n(%)**	**n(%)**	**n(%)**	**n(%)**
**Target population**	People with a decline in functioning/functional ability^e^	2(9.5)	3(17.6)	22(70.9)	20(95.2)
	People with a single health condition	18(85.7)	13(76.5)	8(25.8)	0(0)
	People with more than two health conditions	1(4.8)	1(5.9)	1(3.2)	1(4.8)
**Health condition area**	Cardiovascular	5(27.8)	2(15.4)	1(11.1)	0
	Neurological	3(16.7)	6(46.2)	3(33.3)	0
	Respiratory	3(16.7)	3(23.1)	2(22.2)	0
	Metabolic	2(11.1)	1(7.7)	1(11.1)	0
	Pain	2(11.1)	0	0	0
	Less than 2 studies^f^	3(16.8)	1(7.7)	2(22.2)	0
**Health condition**	COPD	3(16.7)	3(23.1)	2(22.2)	0
	Diabetes	2(11.1)	1(7.7)	0	0
	Heart failure	2(11.1)	1(7.7)	0	0
	Stroke	2(11.1)	6(46.2)	1(11.1)	0
	Cardiovascular disease not specified	2(11.1)	0	1(11.1)	0
	Chronic pain	2(11.1)	0	0	0
	Coronary heart disease	1(5.6)	0	0	0
	Inflammatory arthritis	1(5.6)	0	0	0
	Multiple sclerosis	1(5.6)	0	0	0
	Rotator cuff tendinopathy	1(5.6)	0	0	0
	Cancer not specified	1(5.6)	0	0	0
	Less than 2 studies^g^	0	2(15.4)	5(55.5)	0
**Rehabilitation model**	Community	10(43.5)	10(50)	17(45.9)	4(16)
	Telerehabilitation	8(34.8)	3(15)	6(16.2)	1(4)
	Outpatient	4(17.4)	2(10)	4(10.8)	5(20)
	Home	1(4.4)	5(25)	9(24.3)	10(40)
	Eldercare	0	0	1(2.7)	5(20)
**Health care worker**	Physical therapists	11(28.9)	6(22.2)	10(20.4)	11(22.9)
	Nurses	6(15.8)	6(22.2)	8(16.3)	11(22.9)
	Occupational Therapists	2(5.3)	3(11.1)	5(10.2)	6(12.5)
	Community workers	1(2.6)	3(11.11)	2(4.1)	0
	Dieticians	4(10.5)	1(3.7)	2(4.1)	1(2.1)
	General practitioners (or “family doctors”)	1(2.6)	1(3.7)	2(4.1)	4(8.3)
	Geriatricians	0	1(3.7)	1(2.0)	3(6.3)
	PRM physicians	1(2.6)	0	0	1(2.1)
	Other physicians^h^	2(5.3)	1(3.7)	2(4.1)	1(2.1)
	Social workers	2(5.3)	1(3.7)	2(4.1)	3(6.3)
	Exercise experts	0	1(3.7)	5(10.2)	1(2.1)
	Psychologist	3(7.9)	0	0	0
	Speech and language therapists	1(2.7)	1(3.7)	0	0
	Other^i^	1(2.6)	1(3.7)	2(4.1)	5(10.4)
	Does not applied or not reported	3(7.9)	1(3.7)	8(16.3)	1(2.1)
**Rehabilitation intervention category**	Assessment	11(18.6)	13(27.7)	14(20.6)	16(25.8)
	Care coordination and management	11(18.6)	5(10.6)	12(17.7)	13(20.9)
	Restorative and compensatory approaches	18(30.5)	13(27.7)	26(38.2)	17(27.4)
	Education, counselling and skills training	19(32.2)	14(29.8)	12(17.7)	11(17.7)
	Provision and training in the use of assistive technology	0	1(2.1)	1(1.5)	2(3.2)
	Environmental modifications	0	1(2.1)	3(4.4)	3(4.8)
**Most common rehabilitation interventions**	Therapeutic exercise	8(9.5)	9(13.6)	22(19.5)	11(8.7)
	Education and skills training for selfcare and self-management not specified	14(16.7)	14(21.2)	10(8.9)	5(3.9)
	Assessment person-centred goals	9(10.7)	7(10.6)	6(5.3)	14(11.1)
	Assessment functioning/functional ability	4(4.8)	4(6.1)	7(6.2)	8(6.4)
	Follow up visits	7(8.3)	3(4.6)	6(5.3)	6(4.8)
	Education and skills training for caregivers	3(3.6)	4(6.1)	2(1.8)	6(4.8)
	Education and counselling on behavioural risk factors	4(4.7)	4(6.1)	4(3.5)	1(0.8)
	Education for self-directed therapeutic exercise	4(4.7)	2(3.0)	1(0.9)	4(3.2)
	Monitoring of functioning/functional ability	3(3.6)	1(1.5)	2(1.8)	6(4.8)
	Case management	2(2.4)	1(1.5)	3(2.6)	6(4.8)
	Multicomponent rehabilitation program not specified	1(1.2)	2(3.0)	5(4.4)	2(1.6)
	Motivational interventions	3(3.6)	1(1.5)	4(3.5)	2(1.6)
**Time and intensity of rehabilitation program**					
**Dosage decision**	Adapted	1(4.8)	2( 11.7)	5(16.1)	7(33.3)
	Prespecified	11(52.4)	10( 58.8)	18(58.1)	7(33.3)
	Prespecified and adapted	9(42.9)	5(29.4)	8(25.8)	7(33.3)
		**median(IQR)**	**median(IQR)**	**median(IQR)**	**median(IQR)**
**Median frequency of sessions per week**		1.5(1–2)	1(1–2)	2(0.98–2)	0.46(0.17–2)
**Median session duration in minutes**		90(67.5–120)	120(50–180)	55(40–90)	60(50–65)
**Median number of sessions per patient per program**		10(6–30)	20(18–24)	24(16–43.5)	10(6–16)
**Median rehabilitation length in weeks**		8(6–18)	12(7–17)	20(12–32)	12(9–48)

*COPD* Chronic obstructive pulmonary disease, *COVID-19* Coronavirus disease 2019, *P&RM* Physical and rehabilitation medicine physicians

^a^21 studies (23.3%), mean age 57.8 (SD3.7)

^b^17 studies (18.9%), mean age 67.7(SD2.4)

^c^31 studies (34.4%), mean age 81.77(SD1.76)

^d^21 studies (23.3%), mean age 81.77(1.76)

^e^We considered “functional ability” and “functioning”, as introduced in the International Classification of Functioning, Disability and Health (ICF)(86) as equivalent concepts

^f^Autoimmune disease, Cancer, Musculoskeletal, Communicable diseases

^g^Heart failure, Osteoarthritis, Cognitive Impairment, COVID-19, Sarcopenia

^h^Other physicians included cardiologists and physicians not specified

^i^Other health workers included professional caregivers, home care personnel, dance instructors, welfare officers, pharmacists, mental health workers or therapists not specified, health care managers, music therapists, therapist assistants or students, gerontologists, optometrists

In Table 3, we present results by four age groups labelled “50–60”, “60–70”, “70–80” and “80 + ”. These groups correspond to the four quantiles of the variable “participant’s mean age” (Table 1) and their labels are only approximations of the minimum and maximum age of each group. We found heterogeneity across groups regarding target population, rehabilitation model, rehabilitation interventions, and program dosage. Pronounced differences were observed in the proportion of studies focusing on single health conditions, which decreased with older ages; in the group over 80 + , 95% of the studies focused on limitations in functioning. We did not observe differences concerning healthcare workers providing interventions: PTs, nurses, and OTs remained the most frequent across groups.

### Group 50 to 60 years

Most rehabilitation programs for this group focused on a single health condition, mostly cardiovascular. The community model was the most used (43.5%), followed by telerehabilitation (34.8%). Education and counselling was the most common intervention category (32.5%), closely followed by restorative and compensatory approaches (30.5%). Education and skills training for self-care and self-management was the most common intervention provided (16.7%). The dosage of most rehabilitation programmes in this group was pre-specified and had the lowest number of therapeutic sessions (10 in total) and programme length (8 weeks) compared with other age groups. One exemplary study was conducted in an easily accessible health service of the Norwegian public PC and delivered a group-based chronic pain self-management course with 2.5 h weekly sessions for 6 weeks [58]. Another study used a telecoaching protocol that integrated symptom monitoring with face-to-face video chats with social workers to improve self-management in patients with heart failure [59].

### Group 60 to 70 years

In this group, the focus remained on single health conditions (76.5%); however, the most addressed were of neurological origin (46.2%), with stroke targeted in 6 studies. The most common model used was community (50%), followed by home (25%), and the most common intervention category was education and counselling (29.8%), followed by assessment (27.7%) and restorative and compensatory approaches (27.7%). Most programs were pre-specified and had the highest median session duration (120 min). For instance, a study in the UK combined community and home models to provide rehabilitation for people with stroke; the program included home visits, individualised home-based training, group classes and drop-in sessions, according to the patient’s needs, in a community centre [60]. Another study conducted in China evaluated the effectiveness of a community-based rehabilitation program in increasing the participation in rehabilitation and the functional recovery of stroke survivors [61].

### Group 70 to 80 years

For persons aged 70 to 80 years old programs shifted towards providing rehabilitation to people with or at high risk of functioning decline (70.9%). The most common model used was community (45.9%), followed by home (24.3%). Restorative and compensatory approaches was the most common intervention category (38.2%), and three papers included environmental modifications. This was the group with the greatest emphasis on therapeutic exercise, the most common intervention provided (19.5%). This group received the highest frequency of sessions per week (2 sessions), the highest total number of sessions per patient (24 sessions) and had the longest total programme duration (20 weeks). An exemplary study evaluated whether bathing adaptations at home could prevent deterioration in functioning and reduce the use of other health and social care resources [62]. Another study in Australia used a refurbished local council leisure centre to provide pulmonary and heart failure rehabilitation [63].

### Group older than 80 years

In the 80 + group, all but one study targeted people at risk of or with functional decline. The most frequent delivery model used was home (40%), followed by eldercare and outpatient (20% each). The most common rehabilitation categories were restorative and compensatory approaches (27.4%), followed by assessment (25.8%). The most common intervention was assessment of person-centred goals (11.1%), and correspondently, rehabilitation dosage was very frequently completely or partially adapted to the patient’s needs. This group had the lowest median frequency of sessions, with only one session every two weeks. An exemplary study in Finland investigated the effect of a 12-month home-based exercise program on functioning and the number of falls among persons with frailty signs [64]. Another study in Germany provided home-based therapeutic exercise, motivational interventions, and education on physical activity to older persons with cognitive impairment after discharge from inpatient rehabilitation [65]. In Japan, a study conducted among older persons receiving long-term care at home or at community facilities, provided weekly therapeutic exercise, occupational therapy, and participation in community activities, such as planting flowers in a park, under the supervision of professional caregivers [66].

## Discussion

In this secondary analysis of a previously conducted scoping review [24], we synthesised information from 96 studies, mostly published in HICs, on how rehabilitation services are currently offered in PC for older persons. Rehabilitation services were mostly delivered at community settings but also at home or eldercare facilities as well as through telerehabilitation and outpatient rehabilitation programs. In almost all studies PTs, nurses and OTs were the most common health workers, while task shifting, a typical PC strategy, was reported in only 15.6% of them. The assessment of functioning, coordination of rehabilitation, therapeutic exercise, psychological interventions, and education for self-management were the most frequent rehabilitation interventions provided to older persons in PC, with considerable differences across models. Paradoxically, environmental adaptations as well as the provision and training in the use of assistive technology, which are fundamental rehabilitation interventions and are very relevant to older persons, were rarely reported. Using age groups to compare rehabilitation programs, we observed that the older the participants were, the more programs were focused on functioning decline. In contrast, programs offered for persons between 50 and 70 years old were frequently disease-oriented. The duration and intensity of rehabilitation programs varied significantly across groups. Understanding how rehabilitation services are currently delivered in PC to older persons is highly relevant to the WHO’s current efforts to both strengthen PC to deliver rehabilitation and to achieve the Decade goal of healthy ageing.

Our scoping review expands existing knowledge about models for providing rehabilitation at PC. Our findings show both similarities and differences compared to a previous study by McColl et al. [23], which first described models for integrating rehabilitation and PC services, including clinic, outreach, self-management, community-based rehabilitation (CBR), case management, and shared care. McColl’s clinic model aligns with our outpatient service delivery model, while our home-based, telerehabilitation, and eldercare models can be considered variations of the outreach strategy. Our community model, which refers to health-related rehabilitation services delivered in community settings, differs considerably from McColl’s CBR model. CBR no longer aims solely to improve access to community rehabilitation services but, has evolved into a broader, multi-sectoral approach to community-based inclusive development for people with severe disabilities [67–69]. Both studies identified care coordination and management as key interventions. It is important to stress, however, that the target population is a key difference between our study and McColl’s: while McColl’s study focused on persons with severe impairments and chronic health conditions, ours examined services offered specifically to anyone aged 50+.

To the best of our knowledge, this review is the first to systematically summarise key interventions for rehabilitation offered at PC for persons aged 50 + and our findings are consistent with current trends in research and clinical practice. For example, the WHO’s Integrated Care for Older People (ICOPE) guidelines [70], which are mostly used for the oldest old, also recommend the assessment of functioning and stress the importance of care coordination, emphasising a person-centred approach. Exercise has also been shown to be one of the most important interventions to promote healthy ageing; however, older persons with comorbidities frequently face challenges in accessing and safely engaging in physical activity and therapeutic exercise [71]. Rehabilitation can serve as a key health strategy to ensure that exercise is accessible, safe, and matched to the needs of ageing populations. Furthermore, psychological interventions are essential for older persons, and a growing body of evidence highlights the importance of addressing depression, anxiety, fear of falling, adjustment issues and neurocognitive disorders to optimise the rehabilitation process for older persons [72]. In our study, psychological interventions were indeed a frequent element of rehabilitation programmes. Finally, self-management education, including counselling and skills training for patients and their families, is key to addressing unmet rehabilitation needs resulting from a lack of accessible services, uneven geographic distribution, transportation difficulties and affordability [18] and is frequently delivered to older persons. It is important to stress, however, that rehabilitation is characterised not by a single intervention but by a set of interventions delivered in rehabilitation programs [14]. Currently, the WHO is developing a Basic Rehabilitation Package (BRP) [73], which provides information on low-cost, high-impact, evidence-based rehabilitation interventions that can be delivered by existing PC workers. Given its potential to improve access to rehabilitation for those in need, it would be desirable to assess the extent to which the BRP [73], once implemented, is able to meet the most pressing rehabilitation needs of older persons or whether adjustments would be needed to enhance its potential to foster healthy ageing.

Our findings show that currently, in some HICs, rehabilitation interventions for older persons are being delivered by both traditional rehabilitation workers (e.g., PTs, OTs) and generalist healthcare providers (e.g., nurses, general practitioners). Our results are in line with the WHO’s proposed pathways for integrating rehabilitation into PC [74], which identifies two pathways: in areas with a well-established rehabilitation workforce, rehabilitation workers can be available at PC, while in regions with limited rehabilitation capacity, generalist healthcare providers may take on the responsibility of delivering rehabilitation interventions. In our review, nurses, general practitioners (or “family doctors”), and community workers acted as rehabilitation providers in one-third of the identified studies. Interestingly, they were the sole providers, without the involvement of traditional rehabilitation workers, such as PTs, OTs, or rehabilitation physicians, in approximately one out of four studies. On the other hand, traditional rehabilitation workers provided rehabilitation services at PC without any generalist healthcare providers in almost 40% of studies. Notably, both approaches were employed, and task sharing was still found in 15% of them even though almost all studies included in our review were conducted in HICs. Task shifting or task sharing have been used successfully in other sectors, such as mental health, to address workforce shortages [75, 76]. Shifting and sharing tasks from specialised rehabilitation professionals to PC workers, carers and volunteers enables the expansion of rehabilitation services and reduces the burden on specialised professionals [77], particularly in resource-constrained settings where expansion of the rehabilitation workforce is not economically feasible [78]. It may also be the best strategy to cover unmet needs in the rehabilitation sector in the short term in settings with weak integration of rehabilitation in the health system. The WHO has taken initial steps to address the necessary training, support and mechanisms for task shifting and sharing in rehabilitation by establishing a PC workstream within the World Rehabilitation Alliance [79] and by developing a Rehabilitation Competency Framework [80] that applies to all health workers who provide rehabilitation interventions, not just to traditional rehabilitation workers. However, these strategies do not specifically focus on older persons, which, due to factors like multimorbidity might require more tailored guidance and training for health workers. More research on issues such as the quality and outcomes of task-sharing is needed to develop specific recommendations.

Although our review was quite broad and comprehensive, some key interventions for rehabilitation of the older persons were scarcely reported, particularly the provision of and training in the use of assistive technology and environmental modifications. Assistive technology, such as spectacles, hearing aids, walking canes, wheelchairs, orthoses, or prostheses, is commonly needed by older persons [81]. However, the unmet need for essential assistive technology can be significant, ranging from 2.1% to 83.5% (median: 22.6%) depending on the country and without considering spectacles; access increased with higher Human Development Index (HDI) scores [81]. Incorporating the provision of assistive technology in rehabilitation models provided through PC could help overcome one of the main barriers faced by potential users: limited physical and geographical access [81]. On the other hand, environmental modifications, especially home adaptations, can make a significant contribution to successful ageing at home, allowing older persons to remain in familiar surroundings and maintain wellbeing despite progressive limitations in functioning [82]. The extent to which the aforementioned WHO Basic Rehabilitation Package [73] includes the provision of and training in the use of assistive technology and environmental modifications is a very important criterion in assessing its relevance to ageing populations.

We found that rehabilitation delivery models, interventions and doses of rehabilitation vary considerably according to the chronological age of beneficiaries. However, chronological age may not be the most accurate indicator for determining the type of rehabilitation needed by a person. Acknowledging that population ageing metrics should be built by combining chronological age with the burden of disease experienced by populations (how healthy they are), a study using Global Burden of Disease (GBD) data created a “*global average 65-years-old*” considering a range of age-related diseases and their age-related burden in terms of disability-adjusted life-years (DALYs). The study identified in countries the equivalent chronological age to the “*global average 65-years-old*” and found significant differences: the chronological age equivalent of the “*global average 65-years-old*” ranged from 76.1 years in Japan to 45.6 years in Papua New Guinea [29]. Current studies modelling healthy ageing trajectories [83], for instance investigating the role of multimorbidity [83], also empirically confirm the assumption of the WHO [2] that the ageing process is heterogeneous, leading to different functioning trajectories of persons who may have the same chronological age. Evidence therefore suggests that it may be more appropriate to design and deliver person-centred rehabilitation programmes based on a person’s limitations in functioning, considering existing health conditions, rather than focusing primarily on age as an eligibility criterion, as was observed for people aged 70 + in our review. For example, we show that people aged between 50 and 70 were more likely to receive rehabilitation for a single condition. However, this group has often been shown to have multimorbidity [19] and would therefore benefit from comprehensive approaches that focus on limitations in functioning. A possible reason for the focus on single conditions such as stroke may be that most clinical practice guidelines (CPGs) for rehabilitation are currently disease specific. Indeed, to our knowledge, there is only one functioning-centred guideline that specifically provides guidance on rehabilitation for older persons [84]. Our chronological age analysis can serve as a basis for (re)designing rehabilitation programmes and CPGs that consider multimorbidity, the evidence of heterogeneity in the ageing process and limitations in functioning as a starting point.

### Strength and limitations

Our scoping review has strengths, such as a comprehensive search strategy and a synthesis process using innovative frameworks from rehabilitation and PC. However, it also has limitations. First, the inclusion of only published literature, may not truly represent current practices and should be supplemented with expert knowledge from stakeholder consultations. Second, unfortunately, there is no single definition of PC that can be applied across countries. For this reason, we developed our own operationalisation of a definition for the purposes of this review, but our classification of a rehabilitation programme as a PC only means that that the study met our criteria, not that it was necessarily delivered as part of that country’s PC system. Third, our scoping review focused on describing models rather than assessing their effectiveness, so we cannot suggest superior models or strategies for integrating rehabilitation into PC, nor can we suggest that the identified rehabilitation interventions or programmes were successful in optimising functioning, reducing disability or preventing secondary complications. Fourth, our finding that provision of and training in the use of assistive technology and environmental modifications were scarcely reported may be related to our search strategy, which did not include these terms. Finally, the predominance of HIC studies limits the generalisability of our findings.

## Conclusions

PC can play a key role in assessing functioning and coordinating the rehabilitation process and is also well placed to provide a range of rehabilitation interventions such as therapeutic exercise, psychological interventions, and self-management education. However, there is a notable lack of reporting on key rehabilitation interventions such as the provision of and training in the use of assistive technology and environmental modifications, which could also be provided in PC. By understanding models of rehabilitation service delivery in PC, stakeholders can work towards developing more comprehensive and accessible services that meet the diverse needs of an ageing population, considering multimorbidity, evidence of heterogeneity in the ageing process, and the importance of using limitations in functioning as a starting point. Our findings, which highlight the role of rehabilitation in healthy ageing, are a valuable resource for informing policy, practice, and future research in the context of the Decade. They can also inform the WHO’s ongoing efforts to strengthen PC for the provision of rehabilitation, as recommended by the Rehab2030 initiative and reaffirmed by the recently adopted WHA resolution on strengthening rehabilitation in health systems. However, the conclusions can only be applied to HICs and more studies are needed that reflect the reality of LMICs.

### Supplementary Information

**Additional file 1.** Primary review Protocol.

**Additional file 2.** Protocol Addendum.

**Additional file 3.** PRISMA-ScR_Checklist.

**Additional file 4.** Search strategies.

**Additional file 5.** Included studies’ most important characteristics.

**Additional file 6.** Rehabilitation dosage.

## Data Availability

All data generated or analysed during this study are included in this published article [and its supplementary information files].

## Abbreviations

PHC: Primary Health Care
PC: Primary care
PRIMASYS: Primary health care systems
UN: United Nations
WHO: World Health Organization
WHA: World Health Assembly
The Decade: United Nations Decade of Healthy Ageing
UHC: Universal health coverage
LMICs: Low and Middle-Income Countries
HICs: High-income countries
ICF: International Classification of Functioning, Disability and Health
US: United States
UK: United Kingdom
ADL: Activities of daily living
PTs: Physical therapist
OTs: Occupational therapists
CBR: Community-based rehabilitation
ICOPE: Integrated care for older people
DALYs: Disability-adjusted life-years
