# Correction: Rehabilitation in primary care for an ageing population: a secondary analysis from a scoping review of rehabilitation delivery models

**DOI:** 10.1186/s12913-024-10694-w

**Published:** 2024-02-13

**Authors:** Vanessa Seijas, Roxanne Maritz, Satish Mishra, Renaldo M Bernard, Patricia Fernandes, Viola Lorenz, Barbara Machado, Ana María Posada, Luz Helena Lugo‑Agudelo, Jerome Bickenbach, Carla Sabariego

**Affiliations:** 1 Faculty of Health Sciences and Medicine, University of Lucerne, Alpenquai 4, Lucerne, 6005 Switzerland; 2 Center for Rehabilitation in Global Health Systems (WHO Collaborating Center), Faculty of Health Sciences and Medicine, University of Lucerne, Lucerne, Switzerland; 3 Ageing, Functioning Epidemiology and Implementation, Swiss Paraplegic Research, Nottwil, Switzerland; 4 Disability, Rehabilitation, Palliative and Long‑Term Care, Health Workforce and Service Delivery Unit, Division of Country Health Policies and Systems, WHO Regional Office for Europe, UN City, Marmorvej 51, Copenhagen, 2100 Denmark; 5 Department of Clinical Medicine, Federal University of Parana, R. XV de Novembro, 1299-Centro, Curitiba, PR 80060-000 Brasil; 6 Rehabilitation in Health Research Group, Sede de Investigación Universitaria, University of Antioquia, Cl. 62 # 52-59, Medellín, Colombia

**Correction to**: BMC Health Services Research (2024) 24:123. 10.1186/s12913-023-10387-w.

In this article, the given name and family name of the second author were erroneously transposed: the author name Roxanne Maritz was incorrectly written as Maritz Roxanne.

The author group has been updated above and the original article has been corrected.

