# Supplementary material for: Rehabilitation in primary care for an ageing population: a secondary analysis from a scoping review of rehabilitation delivery models
Source: BMC Health Serv Res. 2024 Jan 23;24:123. doi: 10.1186/s12913-023-10387-w (PMC10804573; doi:10.1186/s12913-023-10387-w)
Supplement: Supplementary file 1 — Additional file 1. Primary review Protocol. [file 12913_2023_10387_MOESM1_ESM.pdf]

Additional file 1

Study Protocol

Rehabilitation delivery models for an ageing population: A scoping review protocol

Keywords: Healthy Aging (Mesh); Aged (Mesh); Rehabilitation (Mesh); Recovery of Function (Mesh); Models, Organizational(Mesh); Health Services for the Aged(Mesh).

Ethical approval: The study did not involve human subjects and thus did not require ethical approval.

Last update: 20 January 2022

Background

The astonishing success of preventive and curative health care services and the global socioeconomic development has led to worldwide population ageing associated with increasing life expectancy and declining fertility rates. However, at the same time, since non-communicable diseases (NCDs) disproportionately affect older people, the world is also facing an epidemiological transition to a higher incidence and prevalence of chronic NCDs[1-3]. A higher morbidity burden leads to a rapid increase in the number of people experiencing disability or declines in functioning, adding pressure to the health and social care systems[4]. These trends urge health policymakers to look for innovative approaches to achieve a not only longer but healthier and more meaningful life.

Healthy life in older age has been called by the World Health Organization in its World Report on Ageing and Health "Healthy Ageing" which is *"the process of developing and maintaining the functional ability that enables well-being in older age."* Functional ability is *"the health-related attributes that enable people to be and to do what they have reason to value, which is made up of the intrinsic capacity of the individual, relevant environmental characteristics, and the interactions between the individual and these characteristics*[2]". The concept of functioning rooted in the WHO International Classification of Functioning, Disability, and Health (ICF)[5] is, in the author's perspective, very similar. Functioning is the *"outcome of complex interactions between the health state of an individual – is determined by health conditions and body functions and structures – and the physical, interpersonal, and social environment*[6]".

Rehabilitation is an essential health service to improve functioning and hence the critical health strategy to achieve healthy ageing[7]. Rehabilitation has shown to be effective in improving functioning; reducing morbidity, including secondary complications, mortality, and health care use, including hospitalization episodes and length of stay; increasing individuals' participation in education, employment, and social life; and extending independent living. Rehabilitation interventions have also been shown to be cost-effective. However, besides its potential, rehabilitation is the health strategy that has

received the least attention in the public's mind and among health policymakers. Rehabilitation is still not a priority of the healthy ageing research and policy agenda. There is a lack of knowledge about providing rehabilitation services to the ageing population. We lack a systematic understanding of how these services should be organized, who should benefit from them, what interventions should be provided, and by whom and how to measure the outcomes of rehabilitation services aiming to foster healthy ageing at the individual and population level.

Aim

To provide an overview of rehabilitation models being used to improve intrinsic capacity or functional ability in the ageing population.

Structured Research Question

- P Ageing population (older than 50 years)
- I Rehabilitation models, strategies, and programs
- C Not applicable
- O Healthy ageing, functioning, functional ability, intrinsic capacity

Conceptual framework

Healthy Ageing

"*Is the process of developing and maintaining the functional ability that enables well-being in older age. Functional ability reflects a person's physical and mental capacities, the environments he or she inhabits and the ways in which people interact with their environment*"[8]

Ageing population

"*Is the shift in the distribution of a country's population toward older ages. An increase in the population's mean or median age, a decline in the fraction of the population composed of children, or a rise in the fraction of the population that is elderly*"[9]

Rehabilitation

"*A set of interventions designed to optimize functioning and reduce disability in individuals with health conditions in interaction with their environment*"[10]

Functioning

The concept of functioning is rooted in the WHO International Classification of Functioning, Disability, and Health (ICF)[5] and is the *"outcome of complex interactions between the health state of an individual – is determined by health conditions and body functions and structures – and the physical, interpersonal, and social environment*[6]".

International Classification of Functioning, Disability and Health (ICF)

« It is a classification of health and health-related domains. As the functioning and disability of an individual occurs in a context, ICF also includes a list of environmental factors. ICF is the WHO framework for measuring health and disability at both individual and population levels. It was officially endorsed by all 191 WHO Member States in the Fifty-fourth World Health Assembly on 22 May 2001(resolution WHA 54.21) as the international standard to describe and measure health and disability. ICF is based on the same foundation as ICD and ICHI and share the same set of extension codes that enable documentation at a higher level of detail"[11]

Model of care

"*Is defined as an evolving conception of how services should be delivered. The evolution of the model of care implies changes to services delivery processes in response, including in the design of care, organization of providers, management of services and continuous performance improvement*"[12]

Settings of care

"*Describe the varied types of arrangements for services delivery, organized further into different facilities, institutions and organizations that provide care. Settings include ambulatory, community, home, in-patient and residential services, whereas facilities refer to infrastructure, such as clinics, health centres, district hospitals, dispensaries or other entities, for example, mobile clinics and pharmacies*"[13]

Types of care

"*Refers to the varied aim of services, such as health protection, health promotion, disease prevention, diagnosis, treatment, management, long-term care, rehabilitation, and*

palliative care, with the specific population intervention and individual services delivered accordingly"[13]

Care coordination

"*A proactive approach in bringing care professionals and providers together around the needs of service users to ensure that people receive integrated and person-focused care across various settings*"[14]

Integrated health services

"*The management and delivery of health services such that people receive a continuum of health promotion, disease prevention, diagnosis, treatment, disease-management, rehabilitation and palliative care services, through the different levels and sites of care within the health system, and according to their needs throughout the life course*"[14]

Integrated health services delivery

"*Is defined as an approach to strengthen people- centred health systems through the promotion of the comprehensive delivery of quality services across the life-course, designed according to the multidimensional needs of the population and the individual and delivered by a coordinated multidisciplinary team of providers working across settings and levels of care. It should be effectively managed to ensure optimal outcomes and the appropriate use of resources based on the best available evidence, with feedback loops to continuously improve performance and to tackle upstream causes of ill health and to promote well-being through intersectoral and multisectoral actions*"[13]

People-centred care

"*An approach to care that consciously adopts individuals', carers', families' and communities' perspectives as participants in, and beneficiaries of, trusted health systems that respond to their needs and preferences in humane and holistic ways. People-centred care also requires that people have the education and support they need to make decisions and participate in their own care. It is organized around the health needs and expectations of people rather than diseases*"[14]

Person-centred care

"Care approaches and practices that see the person as a whole with many levels of needs and goals, with these needs coming from their own personal social determinants of health"[14]

Person-centredness

"Is defined as the extent to which the delivery of services adopts a person-facing perspective, including selecting services according to an individual's needs and known risks, designing care to engage patient's in decision-making, organizing providers to realize their delivery, with management and improvement mechanisms in place towards optimal health outcomes"[13]

People-centred health systems

"are defined as the design of core health system functions that prioritize the needs of individuals, their families and communities, both as participants and beneficiaries for high-quality comprehensive and coordinated services delivered in an equitable manner and involving people as partners in decision-making"[13]

Primary care

"First-contact, accessible, continued, comprehensive and coordinated care to people and communities"[14]

"Describes a type of care and setting for health services delivery that supports first-contact, accessible, continued, comprehensive and coordinated care to individuals and communities"[12]

"Refers to the concept elaborated in the 1978 Declaration of Alma-Ata, which is based on the principles of equity, participation, intersectoral action, appropriate technology and a central role played by the health system"[14]

Primary health care

"Refers to the approach elaborated in the 1978 Declaration of Alma-Ata based on the principles of equity, participation, intersectoral action, appropriate technology and a central role played by the health system for the delivery of services that are made universally accessible to individuals and families in the community through their full participation and at a cost that the community and country can afford to maintain at every stage of their development in the spirit of self-reliance and self-determination"[12]

Primary care facilities

"*Refer to ambulatory care facilities such as primary care centre, office of generalist health professional, ambulatory health care centre, family planning centre, home health care centre, nursing home, and polyclinic; other settings such as walk-in treatment centre, outpatient department of a district/ general hospital, ambulance, mobile clinic, laboratory, pharmacy, and palliative care establishment; and, rural-specific facilities such as rural physician ambulatory, feldscher assistance point, midwifery post and rural health house*"[12]

Long-term care

"*All activities undertaken by others to ensure that people with, or at risk of, a significant ongoing loss of capacity can maintain a level of functional ability consistent with their basic rights, fundamental freedoms and human dignity*"[15]

Residential long-term care facilities

"*Also known as high dependency care facilities, are establishments primarily engaged in providing inpatient nursing and rehabilitative services to individuals requiring nursing care*"[12]

E-health

"*Information and communication technologies that support the remote management of people and communities with a range of health care needs through supporting self-care and enabling electronic communications between health care professionals and patients*"[14]

M-health

"*The use of mobile technologies to support health information and medical practices, often incorporated into services such as health call centres or emergency number services*"[13]

Telehealth

"*The delivery of distance health services, such as remote clinical diagnosis and monitoring, as well as non-clinical functions, including prevention and promotion of health and curative services*"[13]

Health professionals

"*Health professionals (ISCO-08 22) are professionals who establish and undertake research and develop and apply scientific knowledge in a range of health and related fields including: medicine, complementary medicine, dentistry, optometry, environmental health and occupational health. Specific occupations within the classification of health professionals include: physicians, nursing and midwifery professionals; paramedics; opticians; dentists; speech therapists; dieticians; psychiatrists; and, other health professionals. The tasks undertaken by health professionals involve: conducting research and obtaining scientific knowledge through the study of human and animal disorders; diagnosing illnesses and ways of treating them; the planning, management and evaluation of the care of patients; advising on or dispensing and applying preventive and curative measures; promoting health; and, preparing scientific papers and reports*"[12]

Allied health professionals

"*Refers to a diverse group of health care professionals who provide necessary services to patients in addition to, or in place of, services provided by physicians, nurses and paramedical practitioners. Examples include medical technicians, speech therapists, physical therapists, etc*"[12]

Carers (family carers)

"*Refer to individuals who provide unpaid care for a member or members of their family, friends or community (5). They can be any relative (spouse, children, daughter- and son-in-law), friend or neighbour who provides a broad range of assistance with personal care or basic activities of daily living to people with functional limitations. They may provide regular, occasional or routine, 'hands-on' care or be involved in organizing care delivered by others, sometimes even at-distance. Carers can live with or separately from the person receiving care. Carers are in contrast with providers associated with a formal service system, whether paid or on a volunteer basis (formal caregiver)*"[12]

Self-management or self-care

"*The knowledge, skills and confidence to manage one's own health, to care for a specific condition or to recover from an episode of ill health*"[13]

Levels of care in rehabilitation

"*The degree of specialization of care provided by rehabilitation health professionals. Inclusions: Primary, secondary, and tertiary levels of specialization*"[16]

Integration of care in rehabilitation

"*The management of delivering rehabilitation services in conjunction with other health services so that people receive timely, comprehensive and well-coordinated care, according to their needs and across different levels (vertical integration) and along the continuum of care (horizontal integration). Inclusions: Continuum of care, admission and discharge planning, collaboration of health profes- sionals, shared electronic patients' records*"[16]

Patient-centredness in rehabilitation

"*Rehabilitation tailored on the person's needs and provided in partnership with them, their families and communities. Inclusions: Shared decision-making, individual re- habilitation plan, patient, family or other caregiver's education and empowerment, patient family or other caregiver's integration in the rehabilitation process, involvement of peer counsellors, involvement of patients as prosumers*"[16]

International Classification of Health intervention (ICHI)

"*Is a common tool for reporting and analysing health interventions for clinical and statistical purposes. The classification is built around three axes: Target (the entity on which the Action is carried out), Action (a deed done by an actor to a target) and Means (the processes and methods by which the Action is carried out). Extension codes are shared with ICD and ICF in the common foundation and allow users to describe additional detail about the intervention in addition to the relevant ICHI code. A simple, logical syntax links ICHI stem codes and extension codes, interventions performed together, and packages of interventions*"[17]

Universal health coverage (UHC) compendium

« The UHC Compendium is a database of health services and intersectoral interventions designed to assist countries in making progress towards Universal Health Coverage (UHC). It provides a strategic way to organize and present information and creates a framework to think about health services and health interventions.[18]

Methodology

The PRISMA extension for scoping reviews (PRISM ScR) checklist will be used to guide the scoping review's methodology.[19]

Eligibility criteria:

Included publications must describe rehabilitation model targeted at the ageing population and must additionally feature the inclusion criteria documented in **Error! Reference source not found.**.

Table 1. Eligibility criteria

	Inclusion criteria	Exclusion criteria
Population	The research focus is the ageing population	Does not focus on people older than 50 years or, the paper does not describe the population age in the abstract and does not target a health condition prevalent in people older than 50 years.
Scope	Describes a model for providing rehabilitation to the ageing population	Describes needs, functional patterns, disability, risk factors, or protective factors of the ageing population
		Aims to describe or test the effectiveness of a single intervention (e.g., Botulinum toxin for spasticity, exercise for heart failure
		The approach does not include rehabilitation interventions
Outcome	Aims to improve the functioning of the ageing population.	Aims to only improve morbidity, mortality, disease control related outcomes (e.g., serum glucose level, blood pressure, medication use, health services use), interventions adherence, interventions' perceived quality and willingness to continue, enjoyment, participation, health service's use, caregivers burden, implementation barriers or health workers perceptions
Study type	Original research, including, intervention, observational or descriptive studies	Case report, case series, research protocol, letter to the editor, position paper, book or book chapter, narrative review, systematic review, meta-analysis, conference proceeding, grey literature, guideline or retraction letter

Language	Published in English	The full-text study's report is not available or is not available in English.
Publication year	From January 2015 to May 2022	

Information sources and search strategy

At least three authors will work together to build the systematic search strategy. We will conduct the search in indexed databases combining the following three main concepts:

1) Rehabilitation and functioning
2) Models of care or health care approaches
3) The ageing population

The search will include natural language and MeSH terms. We will restrict the search to English. We will not search grey literature, but we will revise the reference list of included studies. The final search strategy will be provided as supplementary material during the results dissemination strategy.

Study selection process

Study selection will be completed in two phases:
- Title and abstract screening: Search results will be imported into an online systematic review software called Rayyan[20]. The inclusion criteria will be integrated into the software as a questionnaire that will be developed a priori. To ensure reliability between reviewers, we will conduct training exercises prior to the formal screening. Teams of two reviewers will use the above eligibility criteria to screen titles and abstracts.

- Full-text screening: Teams of two reviewers will use the same eligibility criteria to screen the full texts of studies in duplicate and independently for eligibility. Similarly, if the decision to exclude or include an article is conflictive, a team meeting will be held with a third team member to make the final decision.

Data extraction:

Data extraction of included papers will be conducted individually and in duplicate. The following information will be extracted using a standardize data extraction form.

→ **General characteristics of the study:** authors, country, publication year, study design, research outcome measures, and paper's conclusion.

→ **Characteristics of the studies' target population:** age-related inclusion criteria, mean age, the number of participants, participants' sex, target population, health condition.

→ **Characteristics of the interventions provided:** data about rehabilitation interventions provided in each study and information about time and intensity.

→ **Characteristics of the rehabilitation services or programs:** level of care, mode of service delivery, organisation of the service provision, including integrated, and information about the providers, including type, team organisation, and role or task shifting or sharing.

Table 2. Variables included in the data extraction form

Variable	Description/instructions
PMID	PMID number if available
Title	Copy and paste from the paper
Publication year	
Journal	
Authors	Enter all author's full name
Abstract	Copy and paste from the paper
Country	Add the country or countries in which the study took place. Use the country's English name, don't use abbreviations, and use capital letters.
Study design	Copy and paste the study design reported in the paper
Target population	Copy and paste the description from the paper
Health condition	Write the health condition's name. If patients with several single health conditions were included, add them separated by a comma without space. For example: diabetes,stroke,hip fracture.<br>If the target population was "patient with multimorbidity," just add multimorbidity. If it was fragility or patients with functional decline, enter not applicable.

Age related-inclusion criteria	Copy and paste the description from the paper
Mean age	Enter the participant's mean age, up to two decimal points. If the mean age was reported only by groups, sum, and divide by the number of groups. For example, if two groups were included, sum the two mean ages, and divide by two.
Number of participants	Enter the total number of participants, including intervention and control group
Gender (%male)	Enter the percentage of male participants. Only up to one decimal point
Description of the intervention	Copy and paste the entire description from the paper
Time and intensity	Enter information like, length of stay or treatment period, intermittent vs continuous treatments, duration of single treatments, number and duration of treatment sessions, and total duration of treatment, service hours.<br>If necessary: add Not reported/No applicable
Paper's conclusion	Copy and paste the conclusion from the abstract
Paper's conclusion category	Select one of the options: 1) Authors found the intervention effective. 2) Authors found the intervention not effective or not different than the usual care. 3) Can't be assessed
Outcomes	If there is more than one outcome, separate them with commas without spaces.<br>Ex: Independence in activities of daily living (Barthel index),Quality of life (SF-36)
Providers	Try to match your finding with any of the following:<br>Health care workers, allied health care workers, peers and volunteers, informal caregivers and family, the patient.<br>If there is more than one provider, separate them by commas with no spaces.<br>Ex: Health care workers, Community health workers
Multidisciplinary rehabilitation team	Enter “Yes” if the at least 3 service providers were involved in the provision of interventions.

Health workers	Try to match your finding with any of the following:<br>Nurse, general practitioner, speech and language therapist, physical therapist, occupational therapists<br>prosthetist & orthotists, PRM Physicians, geriatrician, social workers, psychologist, community-based rehabilitation workers. If necessary, enter additional professions for example: neurologist, geriatricians. If there is more than one provider, separate them by commas with no spaces.<br>Ex: Nurse,Social workers,psychologist
Level of care revised	Select one of the options: Primary health care, specialized health care or multiple levels of care.
Mode of service delivery	Try to match your finding with any of the following:<br>Outpatient, inpatient, home, eldercare institution, telerehabilitation, community<br>If there is more than one mode of service delivery, separate them by commas with no spaces.<br>Ex: outpatient,inpatient,telerehabilitation
Aim healthy ageing?	Enter "Yes" if the papers stated the aim of contributing to healthy ageing
Integrated care?	Enter "Yes" if the paper matched the following definition of Integrated care:<br>Also known as integrated health, coordinated care, comprehensive care, seamless care, or transmural care, is a worldwide trend in health care reforms and new organizational arrangements focusing on more coordinated and integrated forms of care provision.<br>or the WHO definition: "Integrated care is a concept bringing together inputs, delivery, management and organization of services related to diagnosis, treatment, care, rehabilitation and health promotion. Integration is a means to improve services in relation to access, quality, user satisfaction and efficiency[21].
Role or task shifting?	Enter "Yes" if the paper matched the following definition of role or task shifting: a process by which services that are typically delivered by a type of health worker are moved to other with less extensive qualifications or training.[22]

Note: Enter not reported or not applicable accordingly.

Data synthesis:

We will use a descriptive approach for the synthesis and presentation of information on the general characteristics of the studies. The synthesis will include quantitative, (e.g., frequency analysis) of study characteristics, rehabilitation interventions, and rehabilitation services provision (i.e., type of providers, level of care), and qualitative analysis to identify rehabilitation models.

References

1. Vos T, Lim SS, Abbafati C, et al. Global burden of 369 diseases and injuries in 204 countries and territories, 1990–2019: a systematic analysis for the Global Burden of Disease Study 2019. The Lancet 2020;396(10258):1204-22.
2. World Health Organization. World report on ageing and health: World Health Organization 2015.
3. The World Health Organization. World Health Statistics 2022: Monitoring health for the Sustainable Development Goals, 2022.
4. Cieza A, Causey K, Kamenov K, et al. Global estimates of the need for rehabilitation based on the Global Burden of Disease study 2019: a systematic analysis for the Global Burden of Disease Study 2019. The Lancet 2020;396(10267):2006-17. doi: 10.1016/s0140-6736(20)32340-0
5. World Health Organization. International Classification of Functioning, Disability and Health (ICF). Geneva, Switzerland, 2001.
6. Stucki G, Bickenbach J. 1.1 Basic Concepts, Definitions and Models. The Journal of the International Society of Physical and Rehabilitation Medicine 2019;2(5) doi: 10.4103/jisprm.jisprm_5_19
7. Stucki G, Bickenbach J, Gutenbrunner C, et al. Rehabilitation: The health strategy of the 21st century. Journal of rehabilitation medicine : official journal of the UEMS European Board of Physical and Rehabilitation Medicine 2018;50(4):309-16. doi: 10.2340/16501977-2200
8. World Health Organization. Decade of Healthy Ageing Baseline Report: World Health Organization 2020.
9. Weil DN. Population aging: National bureau of economic research Cambridge, Mass., USA, 2006.
10. World Healt Organization. Rehabilitation.

11. World Healt Organization. International Classification of Functioning, Disability and Health (ICF) 2001 [Available from: https://www.who.int/standards/classifications/international-classification-of-functioning-disability-and-health accessed 05.09.2022.
12. World Health Organization, Regional office for Europe. GLOSSARY OF TERMS: WHO European Primary Health Care Impact, Performance and Capacity Tool (PHC-IMPACT). https://www.euro.who.int/__data/assets/pdf_file/0006/421944/Glossary-web-171219.pdf.
13. World Health Organization, Regional office for Europe. Glosary of terms: The European Framework for Action on Integrated Health Services Delivery, 2016.
14. World Health Organization. WHO global strategy on integrated people-centred health services 2016-2026: Placing people and communities at the centre of health services, 2015.
15. Pot AM, Briggs AM, Beard JR, et al. Healthy Ageing and the need for a Long- term-care system: Global consultation on integrated care for older people (ICOPE)–the path to universal health coverage, 2017.
16. Gutenbrunner C, Nugraha B, Gimigliano F, et al. International Classification of Service Organization in Rehabilitation: An updated set of categories (ICSO-R 2.0). Journal of rehabilitation medicine : official journal of the UEMS European Board of Physical and Rehabilitation Medicine 2020;52(1):jrm00004. doi: 10.2340/16501977-2627 [published Online First: 20200102]
17. World Healt Organization. International Classification of Health Interventions (ICHI).
18. World Healt Organization. Universal Health Coverage Compendium
19. Tricco AC, Lillie E, Zarin W, et al. PRISMA extension for scoping reviews (PRISMA-ScR): checklist and explanation. Annals of internal medicine 2018;169(7):467-73.
20. Ouzzani M, Hammady H, Fedorowicz Z, et al. Rayyan—a web and mobile app for systematic reviews. Systematic reviews 2016;5(1):1-10.
21. Gröne O, Garcia-Barbero M. Integrated care: a position paper of the WHO European Office for Integrated Health Care Services. International journal of integrated care 2001;1
22. Organization WH. The world health report 2006: working together for health: World Health Organization 2006.
