# Supplementary material for: Rehabilitation in primary care for an ageing population: a secondary analysis from a scoping review of rehabilitation delivery models
Source: BMC Health Serv Res. 2024 Jan 23;24:123. doi: 10.1186/s12913-023-10387-w (PMC10804573; doi:10.1186/s12913-023-10387-w)
Supplement: Supplementary file 2 — Additional file 2. Protocol Addendum. [file 12913_2023_10387_MOESM2_ESM.pdf]

Additional file 2

Research Protocol Addendum

Secondary analysis focused on Primary Health Care of the scoping review "Rehabilitation delivery models to foster healthy ageing".

Background

Everyone should be able to maintain an optimal level of functional ability throughout the ageing process but large inequities in the functional ability of elderly persons are observed in particular in low- and middle-income countries (LMICs). A study using global burden of disease (GBD) data created an "average global 65-year-old for 2017", considering not only chronological age but also the burden of disease experienced by this average person, a proxy of how healthy or functional a person is at a certain age. The study shows that significant differences by country of residence: the equivalent chronological age to average global 65-year-old spanned from 76.1 years in Japan to 45.6 years in Papua New Guinea(1). In line with this study, the World Health Organization (WHO) baseline report on ageing and health showed that people's ability to meet basic needs depended very much on their country of residence(2). With the objective of optimizing older people's functional ability equitably within and across countries, 2021-2030 was declared by the World Health Assembly (WHA) and the United Nations General Assembly as the Decade of Healthy Ageing (hereby referred to as "*The decade*")(3). Healthy ageing was defined as "*the process of developing and maintaining the functional ability that enables well-being in older age*"(2) while functional ability (FA) is understood as the result of the interaction of a person's intrinsic capacity (IC) and the environment the person lives in(2). The build, social, attitudinal, and political environment – including access to health care that meets the needs of the ageing population – is therefore a key determinant of differences in the functional ability of the ageing population across countries.

Rehabilitation is an essential health strategy to improve functional ability but frequently weakly integrated into health systems. Currently, several efforts are being made to strengthen PHC for the provision of rehabilitation. Rehabilitation is defined as "*a set of interventions designed to optimize*

functioning and reduce disability in individuals with health conditions in interaction with their environment"(12), and has been considered instrumental to moving the healthy ageing agenda forward(13). From a conceptual perspective, "functional ability" and "functioning", as introduced in the International Classification of functioning Disability and Health (ICF)(14) are equivalent. Globally, in 2019, an estimated 2.4 billion people had conditions that would have benefited from rehabilitation; these rehabilitation needs have increased by 63% from 1900 due mainly to population growth and ageing(15). However, most of these needs are still unmet. In LMICs, as much as 50% of people do not receive the rehabilitation they need(16). Aware of the urgency to strengthen health services to provide rehabilitation, the 2023 WHO Executive Board has recommended the World Health Assembly (WHA) pass a resolution about "Strengthening rehabilitation in health systems". If approved, members states will commit, among others, to "*expand rehabilitation to all levels of health, from primary to tertiary, and to ensure the availability and affordability of quality and timely rehabilitation services*"(17).

Given the current global health efforts to strengthen PHC, it is crucial to generate knowledge on how rehabilitation is currently provided to the ageing population within PHC. An influential review identified and characterized models for integrating rehabilitation and primary care, yet it did not focus on the specific needs of the ageing population (22). We conducted a scoping review to identify rehabilitation service delivery models for the ageing population and identified six models: outpatient rehabilitation, telerehabilitation, home rehabilitation, rehabilitation in the community, inpatient rehabilitation, and rehabilitation in eldercare; however, we could not focus on PHC(23). Therefore, the objectives of this secondary analysis of the above mentioned scoping review(23) are a) to describe how rehabilitation services are currently offered in PHC for the ageing population, according to rehabilitation service delivery models, and b) to explore age-related differences in the type of rehabilitation services received. This information is essential for stakeholders and policymakers to (re)design the provision of rehabilitation services at this level, to address the needs of the ageing population.

Methods

For this review, the PCC format (Table 1) will be used to tailor the selection of studies to the research question.

Table 1 PCC format for selecting studies.

Criteria	Determinants
**P**opulation	Elderly people with a decline in functioning (over 50 years)

**C**oncept	Models of care; Rehabilitation services
**C**ontext	Primary Health Care (PHC)

Study Design

We will conduct a secondary analysis of a SR(1) currently under the peer review process. In summary, the primary review, adhering to state-of-the-art methods(2), aimed to provide an overview of rehabilitation service delivery models used to optimise the ageing population's intrinsic capacity and functional ability. In May of 2023, we performed a comprehensive and systematic search in Medline and Embase, incorporating both controlled vocabulary and natural language. The search included three domains: ageing population, rehabilitation, and health services. The most important selection criteria were related to the studies' participants' mean age, higher than 50, and the studies' scope, describing or implementing a model of rehabilitation provision and not the individual testing of specific interventions. Information concerning studies' characteristics, target population; characteristics of the rehabilitation services or programs, including setting, level of care, rehabilitation professionals, interventions provided, and dosage was extracted. The complete methods of the primary SR can be found elsewhere(1).

Eligibility Criteria

In this secondary review, we will apply the same criteria as the primary review but limit the analysis to papers classified as PHC. PHC is defined by WHO as "*a whole-of-society approach to health that aims to maximise the level and distribution of health and well-being through three components: (a) primary care and essential public health functions as the core of integrated health services; (b) multisectoral policies and actions; and (c) empowered people and communities*"(3). We will assess papers for eligibility based on both definitions but will not differentiate between papers that address PHC or PC exclusively. From here on, we will use PHC as it is the more comprehensive term. We will include studies if: the paper self-identifies as PHC, or the rehabilitation interventions are delivered exclusively by PHC workers (e.g., nurses or general practitioners) in a conventional PHC setting (e.g., home or community), and/or the interventions delivered do not necessitate complex equipment or highly specialised training.

Information Sources and Search Strategy

We will utilize the results of the primary review's systematic search strategy, which can be found in detail elsewhere(23) and in the previous research protocol. In summary, we conducted a systematic search in MEDLINE and EMBASE, and supplement this by scanning reference lists of systematic reviews identified during the title and abstract screening process.

Study Selection Process

Expecting a high number of records due to the comprehensive search strategy, a random 35% sample of titles and abstracts was screened independently by two authors in the original scoping review, using Rayyan software(4). Training rounds continued until over 90% agreement was reached, with conflicts resolved by a third author. We will not conduct in this secondary an analysis additional study selection process.

Data Extraction Process

To perform the secondary analysis, we will extract additional data using the WHO's operational framework for primary health care (5), the PRIMASYS approach(6), and the WHO background paper on integrating rehabilitation in PC(7) as conceptual frameworks. The extra data will comprise information on study settings, stakeholder involvement in planning rehabilitation services, and the dosage of rehabilitation programs. This will include specifics on how the dosage of the rehabilitation program was determined, the total length of the program, and the number, duration, and frequency of sessions. To ensure data accuracy, two authors (VL and BM) will independently extract the additional data after successful pilot testing (over 90% agreement).

New variables and definitions:

Variable	Details for data extraction
Co-design of intervention	Describe openly whether patients and/or caregivers were involved in the design of the intervention. This absolutely must take place BEFORE the intervention. Record if their opinion, preferences, etc have been considered and included in the intervention.
Rural or urban setting	Describe whether the intervention took place in the urban or rural area. Enter only the words that have been used in the studies. If no information was provided, then enter "Not reported"
How was the intensity of rehabilitation decided?	Select the most appropriate programme:<br>**Prespecified programme:** The programme was prespecified and no adaptations were possible.<br>**Adapted to the patient's needs:** if the patient/provider was free to choose which interventions to deliver and at what intensity.<br>**Prespecified programme, adapted to the patient's needs:** where the programme was prespecified but could be adapted to patients' needs, resources, and abilities.
Single or multiple types of sessions?	Report if the interventions provided information on single or multiple sessions
Name of the overall intervention	Copy and paste the name given to the program or intervention
Time of the longest session (in minutes)	Report only the time minutes per session that occurred the for the longest time. For example, if psychotherapy lasted 40 minutes and physiotherapy 30 minutes, write 40

Average of session duration (in minutes)	Sum of all individual sessions' duration minutes. For example, Nurse visits 10 minutes, guided physiotherapy 30 minutes, occupational therapy 15 minutes. Add here 18.33
Frequency of the most frequent session (per week)	Report only the frequency per week that occurred most frequently. Write the number (1, 2 or 3 sessions per week) or select= Not reported. Hand calculations are also possible. For example, the paper reported "1 session every two weeks", divide the number of sessions, by the number of weeks, (1/2 = 0.5), 1 session every month, 1/4,=0.25. Other calculations also possible, for example, 1 session every 10 weeks, write 1/10=0.1. Use 4 to calculate the number of weeks for simplicity.
Average of sessions' frequency number (per week)	Sum all the sessions frequencies and divide by the number of sessions for only the frequencies that were reported
Total number of sessions that each patient received	Sum of all individual sessions. For example, 2 Nurse visits, 2 guided physiotherapies, 2 occupational therapies, add here 6
Total duration of the intervention (in weeks)	Report the number of weeks the program lasted or select "Not reported" or "Individualized". Select "Individualized" when the time was adjusted according to the patient's needs. If time is provided in months, multiply by 4 to calculate the number of weeks.

Data Synthesis

New data synthesis will include descriptive quantitative analysis (e.g., frequencies) of study characteristics, interventions, and services provision. It will also incorporate qualitative analysis and an iterative approach for defining characteristics of rehabilitation services and emerging topics.

Patient and Public Involvement Statement and ethical considerations

We will not involve older people, patients or patient representatives in the methodological design, conduct, reporting or dissemination plan of the scoping review. We will not seek ethical approval as this is considered non-human subject's research.

Researchers

Author Name	Department, Institution. City, Country	ORCID ID/Email	Responsibility
Vanessa Seijas (VS)	Faculty of Health Sciences and Medicine University of Lucerne. Lucerne, Switzerland<br><br>Centre for Rehabilitation in Global Health Systems, WHO Collaborating Centre. University of Lucerne. Lucerne, Switzerland<br><br>Ageing, functioning epidemiology and implementation.	0000-0002-2072-8512 vanessa.seijas@paraplegie.ch	Publication guarantor, first and submitting author. Acquisition, analysis, and interpretation of data. Manuscript preparation.

	Swiss Paraplegic Research. Nottwil, Switzerland		
Maritz Roxanne (RM)	Faculty of Health Sciences and Medicine University of Lucerne. Lucerne, Switzerland<br><br>Centre for Rehabilitation in Global Health Systems, WHO Collaborating Centre. University of Lucerne. Lucerne, Switzerland	0000-0002-5711-0388 roxanne.maritz@unilu.ch	Acquisition, analysis, and interpretation of data. Manuscript preparation.
Satish Mishra	Disability, Rehabilitation, Palliative and long-term Care<br>Health Workforce and Service Delivery Unit<br>Division of Country Health Policies and Systems<br>WHO Regional Office for Europe	0000-0002-2520-5892 mishras@who.int	Data interpretation, manuscript revision
Patricia Fernandes (PF)	Department of Clinical Medicine, Federal University of Parana. Parana, Brazil	0000-0002-2106-1852 patriciafernandes.to@gmail.com	Data acquisition and interpretation, manuscript revision
Viola Lorenz (VL)	Faculty of Health Sciences and Medicine University of Lucerne. Lucerne, Switzerland	0000-0003-3176-6642 viola.lorenz@stud.unilu.ch	Data acquisition and interpretation, manuscript revision
Barbara Machado (BM)	Faculty of Health Sciences and Medicine University of Lucerne. Lucerne, Switzerland	0000-0002-5530-4314 barbara.machado@stud.unilu.ch	Data acquisition and interpretation, manuscript revision
Renaldo M Bernard (RB)	Ageing, functioning epidemiology and implementation.<br>Swiss Paraplegic Research. Nottwil, Switzerland	0000-0002-6958-3369 renaldo.bernard@paraplegie.ch	Data acquisition and interpretation, manuscript revision
Ana María Posada (AMP)	Rehabilitation in Health Research group. University of Antioquia. Medellin, Colombia	0000-0002-0727-3269 ana.posadab@udea.edu.co	Data interpretation, manuscript revision
Luz Helena Lugo (LHL)	Rehabilitation in Health Research group. University of Antioquia. Medellin, Colombia	0000-0002-3467-8835 luzh.lugo@gmail.com	Data interpretation, manuscript revision
Jerome Bickenbach (JB)	Faculty of Health Sciences and Medicine University of Lucerne. Lucerne, Switzerland<br><br>Center for Rehabilitation in Global Health Systems, WHO Collaborating Center. University of Lucerne. Lucerne, Switzerland<br><br>Ageing, functioning epidemiology and implementation.<br>Swiss Paraplegic Research. Nottwil, Switzerland	0000-0003-3070-4407 jerome.bickenbach@paraplegie.ch	Data interpretation, manuscript revision

Carla Sabariego (CS)	Faculty of Health Sciences and Medicine University of Lucerne. Lucerne, Switzerland<br><br>Center for Rehabilitation in Global Health Systems, WHO Collaborating Center. University of Lucerne. Lucerne, Switzerland<br><br>Ageing, functioning epidemiology and implementation. Swiss Paraplegic Research. Nottwil, Switzerland	0000-0001-6946-0177 carla.sabariego@paraplegie.ch	Lead investigator. Project supervision. Analysis, and interpretation of data. Manuscript preparation.

References

1. Seijas V, Roxanne M, Benard RM, Fernandes P, Lorenz V, Machado B, et al. Rehabilitation services currently provided in Primary Health Care: A secondary analysis of a scoping review to foster healthy ageing for all [preprint]. Research Square. 2023.
2. Kazi M, Chowdhury N, Chowdhury M, Turin T. Conducting comprehensive scoping reviews to systematically capture the landscape of a subject matter. Population Medicine. 2021;3(December):1-9.
3. Organization WH. A vision for primary health care in the 21st century. Geneva: World Health Organization. 2018.
4. Ouzzani M, Hammady H, Fedorowicz Z, Elmagarmid A. Rayyan—a web and mobile app for systematic reviews. Systematic reviews. 2016;5(1):1-10.
5. Organization WH. Operational framework for primary health care: transforming vision into action. 2020.
6. Sheikh K, Ghaffar A. PRIMASYS: a health policy and systems research approach for the assessment of country primary health care systems. Health Res Policy Syst. 2021;19(1):31.
7. World Health Organisation, Rehabilitation Programme SF, Disability, and Rehabilitation Unit, , Department of Family and Community Medicine at the University of Toronto. Integrating rehabilitation in primary: background paper. 2021.
