# Supplementary material for: Rehabilitation in primary care for an ageing population: a secondary analysis from a scoping review of rehabilitation delivery models
Source: BMC Health Serv Res. 2024 Jan 23;24:123. doi: 10.1186/s12913-023-10387-w (PMC10804573; doi:10.1186/s12913-023-10387-w)
Supplement: Supplementary file 4 — Additional file 4. Search strategies. [file 12913_2023_10387_MOESM4_ESM.pdf]

Additional file 4

Search concepts, terms, and search strategies for each bibliographic database queried.

Table S1. Summary of search concepts and terms

Concepts	Natural language	Medical subject headings
Rehabilitation	functioning, convalescence, Telerehabilitation, restorative, rehabi*, reablement, re–ablement, enablement, "speech therapy", "self management" , “self-management", "self care" , "self-care", "rehabilitation medicine", recover*, "recovery of function", "physical therapy", "physical function", "physical exercise", "patient* recovery", "occupational therapy", "occupational functioning", "language therapy", "intrinsic capacit*", "instrumental ADLs", "instrumental activities of daily living", "healthy aging", "functional status", "Functional recovery", "functional health", "functional gain", "functional autonomy", "functional abilit*", "cognitive function*", "activities of daily living", "active ageing"	Convalescence, Rehabilitation Research, Rehabilitation Centers, Telerehabilitation, Speech Therapy, Self-Management, Self Efficacy, Self Care, Rehabilitation, Recovery of Function, Physical Therapy Modalities, Physical and Rehabilitation Medicine, Occupational Therapy, Language Therapy, International Classification of Functioning, Disability and Health, Independent Living, Healthy Aging, Activities of Daily Living.
Ageing population	frail, fragility, geria*, retir*, seni*, "old age", "physical decline*", "cognitive decline*", "community-dwelling older adults", "function* impairment*", "Functioning decline", ageing, aging, elder*, "older patients", "older adult*", "older person*", "older people"	Aged, 80 and over, Aged, Middle Aged, Frail Elderly, Geriatrics, Age Factors, Sarcopenia
Services	"health planning", "health program", "health service*", "patient care planning", "Community-based", "Community health service", telemonitoring, "transitional care", telehealth, Telerehabilitation, "Long-Term Care", "care model", "team approach", Telecare	Treatment Outcome, Telerehabilitation, Telemedicine, Senior Centers, Program Evaluation, Primary Health Care, Patient Care Team, Patient Care Management, Models, Organizational, Long-Term Care, Home Care Services, Home Care Services, Hospital-Based, Health Services for the Aged, Geriatric Assessment, Delivery of Health Care, Integrated, Comprehensive Health Care, Community Health Nursing, Transitional Care, Quality of Health Care, Program Development, Patient-Centered

		Care, Outpatient Clinics, Hospital, National Health Programs, Health Services, Health Services Needs and Demand, Health Planning, Community Health Services

Table S2. Academic Search MEDLINE search strategy

#	Query	Results
S124	S43 AND S69 AND S122	17,285
S123	S43 AND S69 AND S122	47,440
S122	(S70 OR S71 OR S72 OR S73 OR S74 OR S75 OR S76 OR S77 OR S78 OR S79 OR S80 OR S81 OR S82 OR S83 OR S84 OR S85 OR S86 OR S87 OR S88 OR S89 OR S90 OR S91 OR S92 OR S93 OR S94 OR S95 OR S96 OR S97 OR S98 OR S99 OR S100 OR S101 OR S102 OR S103 OR S104 OR S105 OR S106 OR S107 OR S108 OR S109 OR S110 OR S111 OR S112 OR S113 OR S114 OR S115 OR S116 OR S117 OR S118 OR S119 OR S120 OR S121)	1,459,100
S121	TI functioning OR AB functioning	203,555
S120	TI convalescence OR AB convalescence	5,210
S119	TI Telerehabilitation OR AB Telerehabilitation	1,136
S118	TI restorative OR AB restorative	23,889
S117	TI rehabi* OR AB rehabi*	187,961
S116	TI reablement OR AB reablement	122
S115	TI re–ablement OR AB re–ablement	19
S114	TI enablement OR AB enablement	649
S113	TI "speech therapy" OR AB "speech therapy"	2,659
S112	TI "self management" OR AB "self management" OR TI "self-management" OR AB "self-management"	22,395
S111	TI "self care" OR AB "self care" OR TI "self-care" OR AB "self-care"	21,067
S110	TI "rehabilitation medicine" OR AB "rehabilitation medicine"	1,936
S109	TI recover* OR AB recover*	742,299
S108	TI "recovery of function" OR AB "recovery of function"	2,500
S107	TI "physical therapy" OR AB "physical therapy"	20,119
S106	TI "physical function" OR AB "physical function"	16,488
S105	TI "physical exercise" OR AB "physical exercise"	17,758
S104	TI "patient* recovery" OR AB "patient* recovery"	3,652
S103	TI "occupational therapy" OR AB "occupational therapy"	10,884
S102	TI "occupational functioning" OR AB "occupational functioning"	1,159
S101	TI "language therapy" OR AB "language therapy"	1,396

S100	TI "intrinsic capacit*" OR AB "intrinsic capacit*"	865
S99	TI "instrumental ADLs" OR AB "instrumental ADLs"	203
S98	TI "instrumental activities of daily living" OR AB "instrumental activities of daily living"	4,595
S97	TI "healthy aging" OR AB "healthy aging"	5,560
S96	TI "functional status" OR AB "functional status"	27,844
S95	TI "Functional recovery" OR AB "Functional recovery"	23,888
S94	TI "functional health" OR AB "functional health"	2,277
S93	TI "functional gain" OR AB "functional gain"	621
S92	TI "functional autonomy" OR AB "functional autonomy"	488
S91	TI "functional abilit*" OR AB "functional abilit*"	7,554
S90	TI "cognitive function*" OR AB "cognitive function*"	72,253
S89	TI "activities of daily living" OR AB "activities of daily living"	30,175
S88	TI "active ageing" OR AB "active ageing"	290
S87	(MH "Convalescence")	3,899
S86	(MH "Rehabilitation Research")	232
S85	(MH "Rehabilitation Centers")	8,532
S84	(MH "Telerehabilitation")	754
S83	(MH "Speech Therapy")	6,701
S82	(MH "Self-Management")	4,424
S81	(MH "Self Efficacy")	23,098
S80	(MH "Self Care")	35,221
S79	(MH "Rehabilitation")	18,647
S78	(MH "Recovery of Function")	58,180
S77	(MH "Physical Therapy Modalities")	39,346
S76	(MH "Physical and Rehabilitation Medicine")	3,486
S75	(MH "Occupational Therapy")	14,210
S74	(MH "Language Therapy")	2,040
S73	(MH "International Classification of Functioning, Disability and Health")	775
S72	(MH "Independent Living")	9,537
S71	(MH "Healthy Aging")	1,721
S70	(MH "Activities of Daily Living")	70,033
S69	S44 OR S45 OR S46 OR S47 OR S48 OR S49 OR S50 OR S51 OR S52 OR S53 OR S54 OR S55 OR S56 OR S57 OR S58 OR S59 OR S60 OR S61 OR S62 OR S63 OR S64 OR S65 OR S66 OR S67 OR S68	5,821,140
S68	TI frail OR AB frail	14,773
S67	TI fragility OR AB fragility	18,027

S66	TI geria* OR AB geria*	55,508
S65	TI retir* OR AB retir*	24,285
S64	TI seni* OR AB seni*	65,198
S63	TI "old age" OR AB "old age"	27,502
S62	TI "physical decline*" OR AB "physical decline*"	441
S61	TI "cognitive decline*" OR AB "cognitive decline*"	27,657
S60	TI "community-dwelling older adults" OR AB "community-dwelling older adults"	7,180
S59	TI "function* impairment*" OR AB "function* impairment*"	25,251
S58	TI "Functioning decline" OR AB "Functioning decline"	44
S57	TI ageing OR AB ageing	47,107
S56	TI aging OR AB aging	203,996
S55	TI elder* OR AB elder*	282,288
S54	TI "older patients" OR AB "older patients"	44,253
S53	TI "older adult*" OR AB "older adult*"	99,963
S52	TI "older person*" OR AB "older person*"	12,707
S51	TI "older people" OR AB "older people"	33,134
S50	(MH "Aged, 80 and over")	1,003,407
S49	(MH "Aged")	3,339,004
S48	(MH "Middle Aged")	4,669,969
S47	(MH "Frail Elderly")	13,772
S46	(MM "Geriatrics")	27,118
S45	(MM "Age Factors")	6,297
S44	(MH "Sarcopenia")	7,326
S43	S1 OR S2 OR S3 OR S4 OR S5 OR S6 OR S7 OR S8 OR S9 OR S10 OR S11 OR S12 OR S13 OR S14 OR S15 OR S16 OR S17 OR S18 OR S19 OR S20 OR S21 OR S22 OR S23 OR S24 OR S25 OR S26 OR S27 OR S28 OR S29 OR S30 OR S31 OR S32 OR S33 OR S34 OR S35 OR S36 OR S37 OR S38 OR S39 OR S40 OR S41 OR S42	833,703
S42	TI "health planning" OR AB "health planning"	3,600
S41	TI "health program" OR AB "health program"	5,859
S40	TI "health service*" OR AB "health service*"	120,965
S39	TI "patient care planning" OR AB "patient care planning"	114
S38	TI "Community-based" OR AB "Community-based"	68,095
S37	TI "Community health service" OR AB "Community health service"	503
S36	TI telemonitoring OR AB telemonitoring	1,816
S35	TI "transitional care" OR AB "transitional care"	1,668
S34	TI telehealth OR AB telehealth	7,883

S33	TI Telerehabilitation OR AB Telerehabilitation	1,136
S32	TI "Long-Term Care" OR AB "Long-Term Care"	22,967
S31	TI program	165,422
S30	TI "care model" OR AB "care model"	6,323
S29	TI "team approach" OR AB "team approach"	6,898
S28	TI Telecare OR AB Telecare	710
S27	(MM "Treatment Outcome")	8,065
S26	(MH "Telerehabilitation")	754
S25	(MH "Telemedicine")	33,078
S24	(MH "Senior Centers")	104
S23	(MM "Program Evaluation")	11,451
S22	(MM "Primary Health Care")	54,906
S21	(MM "Patient Care Team")	28,993
S20	(MM "Patient Care Management")	3,018
S19	(MM "Models, Organizational")	6,482
S18	(MH "Long-Term Care")	27,588
S17	(MH "Home Care Services")	35,507
S16	(MM "Home Care Services, Hospital-Based")	1,579
S15	(MH "Health Services for the Aged")	18,137
S14	(MH "Geriatric Assessment")	31,005
S13	(MH "Delivery of Health Care, Integrated")	13,902
S12	(MH "Comprehensive Health Care")	6,747
S11	(MH "Community Health Nursing")	19,737
S10	(MH "Transitional Care")	1,111
S9	(MH "Quality of Health Care")	76,490
S8	(MH "Program Development")	30,183
S7	(MH "Patient-Centered Care")	21,980
S6	(MH "Outpatient Clinics, Hospital")	15,835
S5	(MH "National Health Programs")	33,146
S4	(MH "Health Services")	26,636
S3	(MH "Health Services Needs and Demand")	54,889
S2	(MH "Health Planning")	21,898
S1	(MH "Community Health Services")	32,760
Limiters: Date of Publication: 20150101-20220531; English Language; Human		

Table S3. Academic Search EMBASE search strategy

#	Query	Results
#11	#7 AND #8 AND #9	4,867
#10	#7 AND #8 AND #9	12,197
#9	#3 OR #6	1,626,662
#8	#2 OR #5	898,194
#7	#1 OR #4	917,320
#6	functioning:ti,kw OR convalescence:ti,kw OR telerehabilitation:ab,ti,kw OR restorative:ti,kw OR rehabi*:ab,ti,kw OR reablement:ab,ti,kw OR re–ablement:ab,ti,kw OR enablement:ab,ti,kw OR 'speech therapy':ab,ti,kw OR 'self management':ti,kw OR 'self-management':ti,kw OR 'self care':ti,kw OR 'self-care':ti,kw OR 'rehabilitation medicine':ab,ti,kw OR recover*:ab,ti,kw OR 'recovery of function':ab,ti,kw OR 'physical therapy':ab,ti,kw OR 'physical function':ti,kw OR 'physical exercise':ti,kw OR 'patient*recovery':ti,kw OR 'occupational therapy':ab,ti,kw OR 'occupational functioning':ab,ti,kw OR 'language therapy':ab,ti,kw OR 'intrinsic capacit*':ab,ti,kw OR 'instrumental adls':ab,ti,kw OR 'instrumental activities of daily living':ab,ti,kw OR 'healthy aging':ab,ti,kw OR 'functional status':ti,kw OR 'functional recovery':ab,ti,kw OR 'functional health':ab,ti,kw OR 'functional gain':ab,ti,kw OR 'functional autonomy':ab,ti,kw OR 'functional abilit*':ab,ti,kw OR 'cognitive function*':ti,kw OR 'activities of dailyliving':ab,ti,kw OR 'active ageing':ab,ti,kw	1,469,268
#5	frail:ab,ti,kw OR fragility:ab,ti,kw OR geria*:ab,ti,kw OR retir*:ti,kw OR seni*:ab,ti,kw OR 'old age':ti,kw OR 'physical decline*':ab,ti,kw OR 'cognitive decline*':ab,ti,kw OR 'community-dwelling older adults':ab,ti,kw OR 'function* impairment*':ti,kw OR 'functioning decline':ti,kw OR ageing:ti,kw OR aging:ti,kw OR elder*:ab,ti,kw OR 'older patients':ti,kw OR 'older adult*':ti,kw OR 'older person*':ti,kw OR 'older people':ti,kw	860,667
#4	'health program':ab,ti,kw OR 'health service*':ab,ti,kw OR 'patient care planning':ab,ti,kw OR 'community-based':ab,ti,kw OR 'community health service':ab,ti,kw OR telemonitoring:ab,ti,kw OR 'transional care':ab,ti,kw OR telehealth:ab,ti,kw OR telerehabilitation:ab,ti,kw OR 'long-term care':ab,ti,kw OR program:ti OR 'care model':ab,ti,kw OR 'team approach':ab,ti,kw OR telecare:ab,ti,kw	479,293
#3	'convalescence'/mj OR 'rehabilitation research'/exp OR 'rehabilitation center'/exp OR 'telerehabilitation'/exp OR 'speech therapy'/exp OR 'self care'/mj OR 'rehabilitation'/exp/mj OR 'physiotherapy'/exp OR 'rehabilitation medicine'/exp OR 'occupational therapy'/exp OR 'language therapy'/exp OR 'international classification of functioning, disability and health'/exp OR 'independent living'/exp OR 'healthy aging'/exp OR 'daily life activity'/mj	329,501
#2	'very elderly'/exp/mj OR 'aged'/exp/mj OR 'middle aged'/exp/mj OR 'geriatrics'/exp/mj OR 'sarcopenia'/exp/mj	108,048
#1	treatment outcome'/mj OR 'telerehabilitation'/mj OR 'telemedicine'/mj OR 'senior center'/mj OR 'program evaluation'/mj OR 'primary health care'/mj OR 'patient care'/mj OR 'long term care'/mj OR 'home care'/mj	507,343

	OR 'elderly care'/exp/mj OR 'geriatric assessment'/mj OR 'integrated health care system'/mj OR 'health care'/mj OR 'community health nursing'/mj OR 'transitional care'/mj OR 'health care quality'/mj OR 'program development'/exp/mj OR 'outpatient department'/mj OR 'health service'/mj OR 'health care planning'/mj OR 'community care'/mj
Limiters: Date of Publication: 20150101-20220531; English Language; Human	
