# Supplementary material for: Rehabilitation in primary care for an ageing population: a secondary analysis from a scoping review of rehabilitation delivery models
Source: BMC Health Serv Res. 2024 Jan 23;24:123. doi: 10.1186/s12913-023-10387-w (PMC10804573; doi:10.1186/s12913-023-10387-w)
Supplement: Supplementary file 5 — Additional file 5. Included studies’ most important characteristics. [file 12913_2023_10387_MOESM5_ESM.pdf]

Additional file 5

Table S1. Included studies' publication information

Title	Year	Journal	Authors	Label
Effectiveness of a Technology-Enhanced Integrated Care Model for Frail Older People= A Stepped-Wedge Cluster Randomized Trial in Nursing Homes	2021	Gerontologist	Kim, H. and Y.-i. Jung and G.-S. Kim and H. Choi and Y.-H. Park	Kim, H., 2021
Effects of Adachi Rehabilitation Programme on older adults under long-term care= A multi-centre controlled trial	2021	PloS one	Baba, Y. and C. Ooyama and Y. Tazawa and M. Kohzuki	Baba, Y., 2021
Chronic Pain Self-Management Support With Pain Science Education and Exercise (COMMENCE) for People With Chronic Pain and Multiple Comorbidities= A Randomized Controlled Trial	2020	Archives of physical medicine and rehabilitation	Miller, J. and J. C. MacDermid and D. M. Walton and J. Richardson	Miller, J., 2020
Short-term effect of a chronic pain self-management intervention delivered by an easily accessible primary healthcare service= a randomised controlled trial	2018	BMJ open	Nøst, T. H. and A. Steinsbekk and O. Bratås and K. Grønning	Nøst, T. H., 2018
Twelve-month effect of chronic pain self-management intervention delivered in an easily accessible primary healthcare service - a randomised controlled trial	2018	BMC health services research	Nøst, T. H. and A. Steinsbekk and O. Bratås and K. Grønning	Nøst, T. H., 2018
Influence of the Self-Perception of Old Age on the Effect of a Healthy Aging Program	2018	Journal of clinical medicine	Mendoza-Núñez, V. M. and E. Sarmiento-Salmorán and R. Marín-Cortés and M. d. l. L. Martínez-Maldonado and M. Ruiz-Ramos	Mendoza-Núñez, V. M., 2018
Community Program Improves Quality of Life and Self-Management in Older Adults with Diabetes Mellitus and Comorbidity	2018	Journal of the American Geriatrics Society	Markle-Reid, M. and J. Ploeg and K. D. Fraser and K. A. Fisher and A. Bartholomew and L. E. Griffith and J. Miklavcic and A. Gafni and L. Thabane and R. Upshur	Markle-Reid, M., 2018
The dynamics of self-care in the course of heart failure management= data from the IN TOUCH study	2018	Patient preference and adherence	Lycholip, E. and I. T. Aamodt and I. Lie and T. Simbelyte and R. Puronaite and H. Hillege and A. de Vries and I. Kraai and A. Stromberg and T. Jaarsma and J. Celutkiene	Lycholip, E., 2018
Community-Based Efforts to Prevent and Manage Diabetes in Women Living in Vulnerable Communities	2018	Journal of community health	Lachance, L. and R. P. Kelly and M. Wilkin and J. Burke and S. Waddell	Lachance, L., 2018
Effectiveness of a community-based self-care promoting program for community-dwelling older adults= a randomized controlled trial	2019	Age and ageing	Wong, A. K. C. and F. K. Y. Wong and K. Chang	Wong, A. K. C., 2019
A feasibility study of a mobile phone supported family-centred ADL intervention, F@ce™, after stroke in Uganda	2018	Globalization and health	Kamwesiga, J. T. and G. M. Eriksson and K. Tham and U. Fors and A. Ndiwalana and L. von Koch and S. Guidetti	Kamwesiga, J. T., 2018
Telemedicine-Assisted Self-Management Program for Type 2 Diabetes Patients	2019	Diabetes technology & therapeutics	Von Storch, K. and E. Graaf and M. Wunderlich and C. Rietz and M. C. Polidori and C. Woopen	Von Storch, K., 2019
Integrated primary and geriatric care for frail older adults in the community= Implementation of a complex intervention into real life	2018	European journal of internal medicine	Inzitari, M. and L. M. Pérez and M. B. Enfedaque and L. Soto and F. Díaz and N. Gual and E. Martín and F. Orfila and P. Mulero and R. Ruiz and M. Cesari	Inzitari, M., 2018

Effects of community-based pulmonary rehabilitation in 33 municipalities in Denmark - results from the KOALA project	2018	International journal of chronic obstructive pulmonary disease	Godtfredsen, N. and T. B. Sørensen and M. Lavesen and B. Pors and L. S. Dalsgaard and J. Dollerup and O. Grann	Godtfredsen, N., 2018
Moderators of Treatment Outcomes After Telehealth Self-Management and Education in Adults With Multiple Sclerosis= A Secondary Analysis of a Randomized Controlled Trial	2018	Archives of physical medicine and rehabilitation	Ehde, D. M. and A. Arewasikporn and K. N. Alschuler and A. J. Hughes and A. P. Turner	Ehde, D. M., 2018
Implementation and evaluation of the Norwegian Ullevaal model as a cardiac rehabilitation model in primary care	2019	Disability and rehabilitation	Nilsson B. B. and P. Lunde and I. Holm	Nilsson B. B., 2019
Improving Chronic Disease Self-Management by Older Home Health Patients through Community Health Coaching	2018	International Journal of Environmental Research and Public Health	Dye, C. and D. Willoughby and A.-D. Begum and C. Grady and R. Oran and A. Knudson	Dye, C., 2018
Community-based rehabilitation training after stroke= results of a pilot randomised controlled trial (ReTrain) investigating acceptability and feasibility	2018	BMJ open	Dean, S. G. and L. Poltawski and A. Forster and R. S. Taylor and A. Spencer and M. James and R. Allison and S. Stevens and M. Norris and A. I. Shepherd and P. Landa and R. M. Pulsford and L. Hollands and R. Calitri	Dean, S. G., 2018
Integrated Memory Care Clinic= Design, Implementation, and Initial Results	2018	Journal of the American Geriatrics Society	Clevenger, C. K. and J. Cellar and M. Kovaleva and L. Medders and K. Hepburn	Clevenger, C. K., 2018
Community health service center-based cardiac rehabilitation in patients with coronary heart disease= a prospective study	2017	BMC health services research	Zhang, L. and L. Zhang and J. Wang and F. Ding and S. Zhang	Zhang, L., 2017
Analysis of home-based rehabilitation in patients with motor impairment in primary care= a prospective observational study	2017	BMC Geriatrics	Vega-Ramírez, F. A. and R. López-Liria and G. Granados-Gámez and J. M. Aguilar-Parra and D. Padilla-Góngora	Vega-Ramírez, F. A., 2017
Integrated Care for Older Adults Improves Perceived Quality of Care= Results of a Randomized Controlled Trial of Embrace	2017	JGIM= Journal of General Internal Medicine	Uittenbroek, R. and H. Kremer and S. Spoorenberg and S. Reijneveld and K. Wynia and R. J. Uittenbroek and H. P. H. Kremer and S. L. W. Spoorenberg and S. A. Reijneveld	Uittenbroek, R., 2017
Community-Based Rehabilitation to Improve Stroke Survivors' Rehabilitation Participation and Functional Recovery	2017	American journal of physical medicine & rehabilitation	Ru, X. and H. Dai and B. Jiang and N. Li and X. Zhao and Z. Hong and L. He and W. Wang	Ru, X., 2017
Bathing adaptations in the homes of older adults (BATH-OUT)= Results of a feasibility randomised controlled trial (RCT) 11 Medical and Health Sciences 1117 Public Health and Health Services	2018	BMC Public Health	Whitehead, P. J. and M. R. Golding-Day and S. Belshaw and T. Dawson and M. James and M. F. Walker	Whitehead, P. J., 2018
The Effectiveness of a Proactive Multicomponent Intervention Program on Disability in Independently Living Older People= A Randomized Controlled Trial	2018	Journal of Nutrition, Health & Aging	Van Lieshout, M. R. J. and N. Bleijenberg and M. J. Schuurmans and N. J. de Wit	Van Lieshout, M. R. J., 2018
The effect of telehealth, telephone support or usual care on quality of life, mortality and healthcare utilization in elderly high-risk patients with multiple chronic conditions. A prospective study	2018	Medicina clinica	Valdivieso, B. and A. García-Sempere and G. Sanfélix-Gimeno and R. Faubel and J. Librero and E. Soriano and S. Peiró and G. GeChronic	Valdivieso, B., 2018
The use of case management for community-dwelling older people= the effects on loneliness, symptoms of depression and life satisfaction in a randomised controlled trial	2018	Scandinavian Journal of Caring Sciences	Taube, E. and J. Kristensson and P. Midlöv and U. Jakobsson	Taube, E., 2018
Care Coordination for Community Transitions for Individuals Post-stroke Returning to Low-Resource Rural Communities	2017	Journal of community health	Kitzman, P. and K. Hudson and V. Sylvia and F. Feltner and J. Lovins	Kitzman, P., 2017
Community-based pulmonary rehabilitation in a non-healthcare facility is feasible and effective	2017	Chronic respiratory disease	Cecins, N. and H. Landers and S. Jenkins	Cecins, N., 2017

Patient Characteristics Associated With a Successful Response to Nurse-Led Care Programs Targeting the Oldest-Old= A Comparison of Two RCTs	2017	Worldviews on Evidence-Based Nursing	Bleijenberg, N. and L. Imhof and R. Mahrer-Imhof and M. I. Wallhagen and N. J. Wit and M. J. Schuurmans	Bleijenberg, N., 2017
Impact of a person-centred community rehabilitation service on outcomes for individuals with a neurological condition	2017	Disability and rehabilitation	Barker, R. N. and C. J. Sealey and M. L. Polley and M. C. Mervin and T. Comans	Barker, R. N., 2017
A three-year follow-up of a nurse-led multidisciplinary pulmonary rehabilitation programme in primary health care= a quasi-experimental study	2016	Journal of clinical nursing	Zakrisson, A.-B. and A. Hiyoshi and K. Theander	Zakrisson, A.-B., 2016
Preparation for the generation of the 100-year-old, the development of a wholeness program for brain health in the elderly	2016	Indian Journal of Science and Technology	Young-Mee, K. and H. Hye-Jeon	Young-Mee, K., 2016
Efficacy of an mHealth intervention to stimulate physical activity in COPD patients after pulmonary rehabilitation	2016	The European respiratory journal	Vorrink, S. N. W. and H. S. M. Kort and T. Troosters and P. Zanen and J.-W. J. Lammers	Vorrink, S. N. W., 2016
A Multicomponent Exercise Intervention that Reverses Frailty and Improves Cognition, Emotion, and Social Networking in the Community-Dwelling Frail Elderly= A Randomized Clinical Trial	2016	Journal of the american medical directors association	Tarazona-Santabalbina, F. J. and M. C. Gomez-Cabrera and P. Perez-Ros and F. M. Martinez-Arnau and H. Cabo and K. Tsaparas and A. Salvador-Pascual and L. Rodriguez-Manas and J. Vina	Tarazona-Santabalbina, F. J., 2016
Public health approach to preventing frailty in the community and its effect on healthy aging in Japan	2016	Geriatrics & gerontology international	Shinkai, S.and H. Yoshida and Y. Taniguchi and H. Murayama and M. Nishi and H. Amano and Y. Nofuji and S. Seino and Y. Fujiwara	Shinkai, S, 2016
Multicomponent Program to Reduce Functional Decline in Frail Elderly People= A Cluster Controlled Trial	2016	Journal of the American Board of Family Medicine = JABFM	Ruikes, F. G. H. and S. U. Zuidema and R. P. Akkermans and W. J. J. Assendelft and H. J. Schers and R. T. C. M. Koopmans	Ruikes, F. G. H., 2016
Increasing self-knowledge= Utilizing tele-coaching for patients with congestive heart failure	2016	Social work in health care	Rosen, D. and S. Berrios-Thomas and R. J. Engel	Rosen, D., 2016
Community-based exercise training for people with chronic respiratory and chronic cardiac disease= a mixed-methods evaluation	2016	International journal of chronic obstructive pulmonary disease	McNamara, R. J. and Z. J. McKeough and L. R. Mo and J. T. Dallimore and S. M. Dennis	McNamara, R. J., 2016
Hospital-at-home integrated care programme for older patients with orthopaedic conditions= Early community reintegration maximising physical function	2016	Maturitas	Mas, M. À. and C. Closa and S. J. Santaeugènia and M. Inzitari and A. Ribera and M. Gallofré	Mas, M. À., 2016
A Home- and Community-Based Physical Activity Program Can Improve the Cardiorespiratory Fitness and Walking Capacity of Stroke Survivors	2016	Journal of stroke and cerebrovascular diseases = the official journal of National Stroke Association	Marsden, D. L. and A. Dunn and R. Callister and P. McElduff and C. R. Levi and N. J. Spratt	Marsden, D. L., 2016
The effects of a pro-active integrated care intervention for frail community-dwelling older people= A quasi-experimental study with the GP-practice as single entry point Health services research	2016	BMC Geriatrics	Looman, W. M. and I. N. Fabbricotti and R. De Kuyper and R. Huijsman	Looman, W. M., 2016
A self-managed single exercise programme versus usual physiotherapy treatment for rotator cuff tendinopathy= a randomised controlled trial (the SELF study)	2016	Clinical rehabilitation	Littlewood, C. and M. Bateman and K. Brown and J. Bury and S. Mawson and S. May and S. J. Walters	Littlewood, C., 2016
A pilot evaluation of Arthritis Self-Management Program by lay leaders in patients with chronic inflammatory arthritis in Hong Kong	2016	Clinical rheumatology	Leung, Y.-Y. and J. Kwan and P. Chan and P. K. K. Poon and C. Leung and L.-S. Tam and E. K. Li and A. Kwok	Leung, Y.-Y., 2016

Effects of an Updated Preventive Home Visit Program Based on a Systematic Structured Assessment of Care Needs for Ambulatory Frail Older Adults in Japan= A Randomized Controlled Trial	2016	The journals of gerontology. Series A, Biological sciences and medical sciences	Kono, A. and K. Izumi and N. Yoshiyuki and Y. Kanaya and L. Z. Rubenstein	Kono, A., 2016
Reablement in community-dwelling older adults= a cost-effectiveness analysis alongside a randomized controlled trial	2016	Health Economics Review	Kjerstad, E. and H. K. Tuntland	Kjerstad, E., 2016
Feasibility study of an integrated stroke self-management programme= a cluster-randomised controlled trial	2016	BMJ open	Jones, F. and H. Gage and A. Drummond and A. Bhalla and R. Grant and S. Lennon and C. McKevitt and A. Riazi and M. Liston	Jones, F., 2016
A randomized controlled trial of telephone-mentoring with home-based walking preceding rehabilitation in COPD	2016	International journal of chronic obstructive pulmonary disease	Cameron-Tucker, H. L. and R. Wood-Baker and L. Joseph and J. A. Walters and N. Schüz and E. H. Walters	Cameron-Tucker, H. L., 2016
Effectiveness of adaptive physical activity combined with therapeutic patient education in stroke survivors at twelve months= a non-randomized parallel group study	2016	European journal of physical and rehabilitation medicine	Calugi, S. and M. Taricco and P. Rucci and S. Fugazzaro and M. Stuart and L. Dallolio and P. Pillastrini and M. P. Fantini	Calugi, S., 2016
Effectiveness of a Proactive Primary Care Program on Preserving Daily Functioning of Older People= A Cluster Randomized Controlled Trial	2016	Journal of the American Geriatrics Society	Bleijenberg, N. and I. Drubbel and M. J. Schuurmans and H. t. Dam and N. P. A. Zuithoff and M. E. Numans and N. J. Wit	Bleijenberg, N., 2016
Lessons learnt from a cluster-randomised trial evaluating the effectiveness of Self-Management Support (SMS) delivered by practice nurses in routine diabetes care	2015	BMJ open	Van Dijk-de Vries, A. and M. A. van Bokhoven and B. Winkens and B. Terluin and J. A. Knottnerus and T. van der Weijden and J. T. M. van Eijk	Van Dijk-de Vries, A., 2015
It's LiFe! Mobile and Web-Based Monitoring and Feedback Tool Embedded in Primary Care Increases Physical Activity= A Cluster Randomized Controlled Trial	2015	Journal of medical Internet research	Van der Weegen, S. and R. Verwey and M. Spreeuwenberg and H. Tange and T. van der Weijden and L. de Witte	Van der Weegen, S., 2015
An Integrated Model of Co-ordinated Community-Based Care	2015	The Gerontologist	Scharlach, A. E. and C. L. Graham and C. Berridge	Scharlach, A. E., 2015
Skill-sharing between allied health professionals in a community setting= A randomised controlled trial	2015	International journal of therapy and rehabilitation	Pighills, A. C. and M. Bradford and K. Bell and L. J. Flynn and G. Williams and D. Hornsby and D. J. Torgerson and M. Kaltner	Pighills, A. C., 2015
Effects of community based cardiac rehabilitation= Comparison with a hospital-based programme	2015	European journal of cardiovascular nursing	Mosleh, S. M. and C. M. Bond and A. J. Lee and A. Kiger and N. C. Campbell	Mosleh, S. M., 2015
Community-based walking exercise for peripheral artery disease= An exploratory pilot study	2015	Vascular medicine (London, England)	Mays, R. J. and W. R. Hiatt and I. P. Casserly and R. K. Rogers and D. S. Main and W. M. Kohrt and P. M. Ho and J. G. Regensteiner	Mays, R. J., 2015
A COPD health management program in a community-based primary care setting= a randomized controlled trial	2015	Respiratory care	Lou, P. and P. Chen and P. Zhang and J. Yu and Y. Wang and N. Chen and L. Zhang and H. Wu and J. Zhao	Lou, P., 2015
Development and evaluation of a nurse-led, tailored stroke self-management intervention	2015	BMC health services research	Kidd, L. and M. Lawrence and J. Booth and A. Rowat and S. Russell	Kidd, L., 2015
OPTIMAL, an occupational therapy led self-management support programme for people with multimorbidity in primary care= a randomized controlled trial	2015	BMC family practice	Garvey, J. and D. Connolly and F. Boland and S. M. Smith	Garvey, J., 2015
Cluster Randomized Controlled Trial= Clinical and Cost-Effectiveness of a System of Longer-Term Stroke Care	2015	Stroke	Forster, A. and J. Young and K. Chapman and J. Nixon and A. Patel and I. Holloway and K. Mellish and S. Anwar and R. Breen and M. Knapp and J. Murray and A. Farrin	Forster, A., 2015

Effects of a community-based multimodal exercise program on physical function and quality of life in cancer survivors= a pilot study	2015	Physiotherapy theory and practice	Foley, M. P. and V. A. Barnes and S. M. Hasson	Foley, M. P., 2015
Development and feasibility testing of an education program to improve knowledge and self-care among Aboriginal and Torres Strait Islander patients with heart failure	2015	Rural and remote health	Clark, R. A. and B. Fredericks and N. J. Buitendyk and M. J. Adams and J. Howie-Esquivel and K. A. Dracup and N. M. Berry and J. Atherton and S. Johnson	Clark, R. A., 2015
Comparing the effects of a home-based exercise program using a gerontechnology to a community-based group exercise program on functional capacities in older adults after a or injury	2018	Experimental gerontology	Martel, D. and M. LauzŽ and A. Agnoux and L. F. d. Laclos and R. Daoust and M. ƒmond and M. J. Sirois and M. Aubertin-Leheudre	Martel, D., 2018
Pre-post effectiveness evaluation of Chronic Disease Self-Management Program (CDSMP) participation on health, well-being and health service utilization	2020	Chronic illness	Hevey, D. and J. W. O'Raghallaigh and V. O. Doherty and K. Lonergan	Hevey, D., 2020
Implementation of a gerontology nurse specialist role in primary health care= Health professional and older adult perspectives	2018	J Clin Nurs	King, A. I. I. and M. L. Boyd and L. Dagley and D. L. Raphael	King, A. I. I., 2018
Improving Health Related Quality of Life and Independence in Community Dwelling Frail Older Adults through a Client-Centred and Activity-Oriented Program. A Pragmatic Randomized Controlled Trial	2016	J Nutr Health Aging	De Vriendt, P. and W. Peersman and A. Florus and M. Verbeke and D. Van de Velde	De Vriendt, P., 2016
The effect of group exercises on balance, mobility, and depressive symptoms in older adults with mild cognitive impairment= a randomized controlled trial	2019	Clin Rehabil	Langoni, C. D. S. and T. L. Resende and A. B. Barcellos and B. Cecchele and J. N. da Rosa and M. S. Knob and T. D. N. Silva and T. S. Diogo and I. G. da Silva and C. H. A. Schwanke	Langoni, C. D. S., 2019
Reablement in a community setting	2015	Disabil Rehabil	Winkel, A. and H. Langberg and E. E. Waehrens	Winkel, A., 2015
The Home Independence Program with non-health professionals as care managers= an evaluation	2016	Clinical Interventions in Aging	Lewin, G. and K. Concanen and D. Youens	Lewin, G., 2016
Reducing disability in community-dwelling frail older people= cost-effectiveness study alongside a cluster randomised controlled trial	2015	Age Ageing	Metzelthin, S. F. and E. van Rossum,M. R. Hendriks,L. P. De Witte,S. O. Hobma,W. Sipers,G. I. Kempen	Metzelthin, S. F., 2015
The Effect and Adherence of a Volunteer-Led Community Care Station Program on Physical Function among Community Older Adults= A One-Year Observational Study	2022	International Journal of Gerontology	Liang, C. C. and Chang, Q. X. and Chen, J. C.	Liang, C. C., 2022
Effect of a physical activity and behaviour maintenance programme on functional mobility decline in older adults= the REACT (Retirement in Action) randomised controlled trial	2022	Lancet Public Health	Stathi, A. and Greaves, C. J. and Thompson, J. L. and Withall, J. and Ladlow, P. and Taylor, G. and Medina-Lara, A. and Snowsill, T. and Gray, S. and Green, C. and Johansen-Berg, H. and Sexton, C. E. and Bilzon, J. L. J. and deKoning, J. and Bollen, J. C. and Moorlock, S. J. and Western, M. J. and Demnitz, N. and Seager, P. and Guralnik, J. M. and Rejeski, W. J. and Hillsdon, M. and Fox, K. R.	Stathi, A., 2022
Effectiveness of a Telerehabilitative Home Exercise Program on Elder Adults' Physical Performance, Depression and Fear of Falling	2022	Percept Mot Skills	Tekin, F. and Cetisli-Korkmaz, N.	Tekin, F., 2022
Development of a Videogame for the Promotion of Active Aging Through Depression Prevention, Healthy Lifestyle Habits, and Cognitive Stimulation for Middle-to-Older Aged Adults	2021	Games Health J	Otero, P. and Cotardo, T. and Blanco, V. and Vazquez, F. L.	Otero, P., 2021
Effects of a Physical Exercise Program on Physiological, Psychological, and Physical Function of Older Adults in Rural Areas	2021	Int J Environ Res Public Health	Kim, S. and Lee, E. J. and Kim, H. O.	Kim, S., 2021

The Effect of Group Music Therapy with Physical Activities to Prevent Frailty in Older People Living in the Community	2021	Int J Environ Res Public Health	Sun, F. C. and Li, H. C. and Wang, H. H.	Sun, F. C., 2021
Impact of Different Aquatic Exercise Programs on Body Composition, Functional Fitness and Cognitive Function of Non-Institutionalized Elderly Adults= A Randomized Controlled Trial	2021	Int J Environ Res Public Health	Farinha, C. and Teixeira, A. M. and Serrano, J. and Santos, H. and Campos, M. J. and Oliveiros, B. and Silva, F. M. and Cascante-Rusenhack, M. and Luis, P. and Ferreira, J. P.	Farinha, C., 2021
Home-based rehabilitation programme compared with traditional physiotherapy for patients at risk of poor outcome after knee arthroplasty= the CORKA randomised controlled trial	2021	BMJ Open	Barker, K. L. and Room, J. and Knight, R. and Dutton, S. and Toye, F. and Leal, J. and Kenealy, N. and Maia Schlussel, M. and Collins, G. and Beard, D. and Price, A. J. and Underwood, M. and Drummond, A. and Lamb, S. and group, Corka Trial	Barker, K. L., 2021
Patient characteristics and outcome in three different working models of home-based rehabilitation= a longitudinal observational study in primary health care in Norway	2021	BMC Health Serv Res	Meisingset, I. and Bjerke, J. and Taraldsen, K. and Gunnes, M. and Sand, S. and Hansen, A. E. and Myhre, G. and Evensen, K. A. I.	Meisingset, I., 2021
Effect of 12-Month Supervised, Home-Based Physical Exercise on Functioning Among Persons With Signs of Frailty= A Randomized Controlled Trial	2021	Arch Phys Med Rehabil	Suikkanen, S. and Soukkio, P. and Aartolahti, E. and Kaaria, S. and Kautiainen, H. and Hupli, M. T. and Pitkala, K. and Sipila, S. and Kukkonen-Harjula, K.	Suikkanen, S., 2021
Remote Home-Based Exercise Program to Improve the Mental State, Balance, and Physical Function and Prevent Falls in Adults Aged 65 Years and Older During the COVID-19 Pandemic in Seoul, Korea	2021	Med Sci Monit	Yi, D. and Yim, J.	Yi, D., 2021
Effects of a home-based physical training and activity promotion program in community-dwelling older persons with cognitive impairment after discharge from rehabilitation= A randomized controlled trial	2022	J Gerontol A Biol Sci Med Sci	Ullrich, P. and Werner, C. and Schonstein, A. and Bongartz, M. and Eckert, T. and Beurskens, R. and Abel, B. and Bauer, J. M. and Lamb, S. E. and Hauer, K.	Ullrich, P., 2022
Multilevel mHealth Intervention Increases Physical Activity of Older Adults Living in Retirement Community	2021	J Phys Act Health	Hosteng, K. R. and Simmering, J. E. and Polgreen, L. A. and Cremer, J. F. and Segre, A. M. and Francis, S. L. and Whitaker, K. M. and Polgreen, P. M. and Carr, L. J.	Hosteng, K. R., 2021
Effect of Home-Based Exercise Program on Physical Function and Balance in Older Adults With Sarcopenia= A Multicenter Randomized Controlled Study	2021	J Aging Phys Act	Sen, E. I. and Eyigor, S. and Dikici Yagli, M. and Ozcete, Z. A. and Aydin, T. and Kesiktas, F. N. and Aydin, F. Y. and Vural, M. and Sahin, N. and Karan, A.	Sen, E. I., 2021
Effects of Cognitive/Exercise Dual-Task Program on the Cognitive Function, Health Status, Depression, and Life Satisfaction of the Elderly Living in the Community	2021	Int J Environ Res Public Health	Sok, S. and Shin, E. and Kim, S. and Kim, M.	Sok, S., 2021
Effects of an Information and Communication Technology-Based Fitness Program on Strength and Balance in Female Home Care Service Users	2021	Int J Environ Res Public Health	Jungreitmayr, S. and Ring-Dimitriou, S. and Trukeschitz, B. and Eisenberg, S. and Schneider, C.	Jungreitmayr, S., 2021
Efficacy of Multidomain Intervention Against Physio-cognitive Decline Syndrome= A Cluster-randomized Trial	2021	Arch Gerontol Geriatr	Liang, C. K. and Lee, W. J. and Hwang, A. C. and Lin, C. S. and Chou, M. Y. and Peng, L. N. and Lin, M. H. and Chen, L. K.	Liang, C. K., 2021
A Pilot Study of a Group Program Focused on Enabling Life Performance for Older Adults Living in the Community	2022	Int J Environ Res Public Health	Furuta, K. and Kobayashi, N. and Kobayashi, R. and Ishibashi, H. and Ishibashi, Y.	Furuta, K., 2022
Effects of rural community-based integrated exercise and health education programs on the mobility function of older adults with knee osteoarthritis	2021	Aging Clin Exp Res	Oh, S. L. and Kim, D. Y. and Bae, J. H. and Lim, J. Y.	Oh, S. L., 2021

Effectiveness of Community-Delivered Functional Power Training Program for Frail and Pre-frail Community-Dwelling Older Adults= a Randomized Controlled Study	2021	Prev Sci	Tou, N. X. and Wee, S. L. and Seah, W. T. and Ng, D. H. M. and Pang, B. W. J. and Lau, L. K. and Ng, T. P.	Tou, N. X., 2021
Contribution of a multicomponent intervention on functional capacity and independence on activities of daily living in individuals with neurocognitive disorder	2021	BMC Geriatr	Borges-Machado, F. and Barros, D. and Teixeira, L. and Ribeiro, O. and Carvalho, J.	Borges-Machado, F., 2021
Effects of multicomponent exercise training intervention on hemodynamic and physical function in older residents of long-term care facilities= A multicenter randomized clinical controlled trial	2021	J Bodyw Mov Ther	Pepera, G. and Christina, M. and Katerina, K. and Argirios, P. and Varsamo, A.	Pepera, G., 2021
Impact of the HOP-UP-PT program on older adults at risk to fall= a randomized controlled trial	2021	BMC Geriatr	Arena, S. K. and Wilson, C. M. and Boright, L. and Peterson, E.	Arena, S. K., 2021
Multi-component cognitive intervention for older adults with mixed cognitive levels= implementation and preliary effectiveness in real-world settings	2021	BMC Geriatr	Mao, H. F. and Tsai, A. Y. and Chang, L. H. and Tsai, I. L.	Mao, H. F., 2021
Reversing the deconditioning effects of the pandemic in the elderly via telerehabilitation	2021	Neurological Sciences and Neurophysiology	Bagkur, M. and Yerlikaya, T. and Inanc, G. and Oniz, A.	Bagkur, M., 2021
An Integrated Model of Community Care for Older Adults= Design, Feasibility and Evaluation of Impact and Sustainability	2021	Aging Medicine and Healthcare	Woo, J. and Yu, R. and Leung, G. and Chiu, C. and Hui, A. and Ho, F.	Woo, J., 2021

Table S2. Included studies' most important characteristics

First author, year	Study design	Country	Rural or urban setting	Target population	Health condition	Health condition area	Inclusion criterion in years	N°of participants*	Participants' mean age**	Participants' sex predominance***
Kim, H., 2021	Randomized controlled trial (Intervention study)	South Korea	_	Fragility or functional decline	_	_	_	913	82.9	Female predominance
Baba, Y., 2021	Randomized controlled trial (Intervention study)	Japan	Urban	Fragility or functional decline	_	_	> 65	78	83.65	Female predominance
Miller, J., 2020	Randomized controlled trial (Intervention study)	Canada	_	Multimorbidity	_	_	_	102	52.8	Female predominance
Nøst, T. H., 2018	Randomized controlled trial (Intervention study)	Norway	Urban	Single health condition	Chronic pain	Pain	> 18	121	52.7	Female predominance
Nøst, T. H., 2018	Randomized controlled trial (Intervention study)	Norway	Urban	Single health condition	Chronic pain	Pain	> 18	121	53	Female predominance
Mendoza-Núñez, V. M., 2018	Pretest-posttest (Intervention study)	Mexico	_	Fragility or functional decline	_	_	_	64	66	Female predominance
Markle-Reid, M., 2018	Randomized controlled trial (Intervention study)	Canada	_	Multimorbidity	_	_	> 65	159	_	Balanced
Lycholip, E., 2018	Randomized controlled trial (Intervention study)	Netherlands	_	Single health condition	Heart failure	Cardiovascular	> 18	118	68	Male predominance
Lachance, L., 2018	Cohort study (Observational Study)	United States of America	_	Single health condition	Diabetes	Metabolic	> 18	161	60.35	_
Wong, A. K. C., 2019	Randomized controlled trial (Intervention study)	China (Hong Kong)	_	Fragility or functional decline	_	_	> 60	457	78	Female predominance

Kamwesiga, J. T., 2018	Quasi-experimental trial (Intervention study)	Uganda	Rural	Single health condition	Stroke	Neurological	> 18	28	59.85	Female predominance
Von Storch, K., 2019	Quasi-experimental trial (Intervention study)	Germany	_	Single health condition	Diabetes	Metabolic	Other	115	58.9	Balanced
Inzitari, M., 2018	Pretest-posttest (Intervention study)	Spain	Urban	Fragility or functional decline	_	_	Other	185	81.6	Female predominance
Godtfredsen, N., 2018	Cohort study (Observational Study)	Denmark	_	Single health condition	COPD	Respiratory	_	581	68.1	Balanced
Ehde, D. M., 2018	Randomized controlled trial (Intervention study)	United States of America	_	Single health condition	Multiple sclerosis	Neurological	> 18	163	52.2	_
Nilsson B. B., 2019	Cohort study (Observational Study)	Norway	_	Single health condition	Cardiovascular disease not specified	Cardiovascular	_	273	56	Male predominance
Dye, C., 2018	Quasi-experimental trial (Intervention study)	United States of America	Rural	Multimorbidity	_	_	> 60	53	_	_
Dean, S. G., 2018	Pilot randomized controlled trial (Intervention study)	United Kingdom	_	Single health condition	Stroke	Neurological	> 18	45	70.5	Male predominance
Clevenger, C. K., 2018	Model development (Descriptive study)	United States of America	_	Single health condition	Cognitive Impairment including dementia or aphasia	Neurological	_	119	78.6	Female predominance
Zhang, L., 2017	Randomized controlled trial (Intervention study)	China	Urban	Single health condition	Coronary heart disease	Cardiovascular	< 75	126	63.1	Male predominance
Vega-Ramírez, F. A., 2017	Cohort study (Observational Study)	Spain	_	Fragility or functional decline	_	_		473	83	Balanced
Uittenbroek, R., 2017	Randomized controlled trial (Intervention study)	Netherlands	_	Fragility or functional decline	_	_	> 75	1456	80.75	Balanced
Ru, X., 2017	Quasi-experimental trial (Intervention study)	China	_	Single health condition	Stroke	Neurological	_	964	68.95	Balanced
Whitehead, P. J., 2018	Randomized controlled trial (Intervention study)	United Kingdom	_	Fragility or functional decline	_	_	> 65	60	77	Balanced
Van Lieshout, M. R. J., 2018	Randomized controlled trial (Intervention study)	Netherlands	Rural	Fragility or functional decline	_	_	> 65	281	74	Balanced
Valdivieso, B., 2018	Randomized controlled trial (Intervention study)	Spain	Urban	Multimorbidity	_	_	> 18	472	73.08	Balanced
Taube, E., 2018	Randomized controlled trial (Intervention study)	Sweden	Rural	Fragility or functional decline	_	_	> 65	153	81.5	Female predominance
Kitzman, P., 2017	Pretest-posttest (Intervention study)	United States of America	Rural	Single health condition	Stroke	Neurological	_	30	65	Balanced

Cecins, N., 2017	Cohort study (Observational Study)	Australia	_	Single health condition	COPD	Respiratory	_	251	72	Balanced
Bleijenberg, N., 2017	Randomized controlled trial (Intervention study)	Netherlands Switzerland	_	Fragility or functional decline	_	_	Other	1791	85.05	Female predominance
Barker, R. N., 2017	Cohort study (Observational Study)	Australia	_	Fragility or functional decline	_	_	Other	206	61	Balanced
Zakrisson, A.-B., 2016	Quasi-experimental trial (Intervention study)	Sweden	Urban,Rural	Single health condition	COPD	Respiratory	_	103	67.7	Balanced
Young-Mee, K., 2016	Quasi-experimental trial (Intervention study)	Korea	_	Fragility or functional decline	_	_	> 65	20	_	Not reported/No applicable
Vorrink, S. N. W., 2016	Randomized controlled trial (Intervention study)	Netherlands	_	Single health condition	COPD	Respiratory	Other	157	62.5	Balanced
Tarazona-Santabalbina, F. J., 2016	Randomized controlled trial (Intervention study)	Spain	Rural	Fragility or functional decline	_	_	> 70	100	80	Balanced
Shinkai, S, 2016	Cohort study (Observational Study)	Japan	_	Fragility or functional decline	_	_	> 65		_	_
Ruikes, F. G. H., 2016	Cluster controlled trial (Intervention study)	Netherlands	_	Fragility or functional decline	_	_	> 70	536	81.8	Female predominance
Rosen, D., 2016	Pretest-posttest (Intervention study)	United States of America	Urban	Single health condition	Heart failure	Cardiovascular	Other	45	60	Female predominance
McNamara, R. J., 2016	Intervention study	Australia	_	Single health condition	COPD, Cardiovascular disease not specified	Cardiovascular, Respiratory	_	32	72	Male predominance
Mas, M. À., 2016	Cohort study (Observational Study)	Spain	Urban	Fragility or functional decline	_	_	> 65	270	83.5	Female predominance
Marsden, D. L., 2016	Pilot randomized controlled trial (Intervention study)	Australia	_	Single health condition	Stroke	Neurological	> 18	20	58.2	Female predominance
Looman, W. M., 2016	Quasi-experimental trial (Intervention study)	Netherlands	_	Fragility or functional decline	_	_	> 75	377	82.05	Female predominance
Littlewood, C., 2016	Randomized controlled trial (Intervention study)	United Kingdom	_	Single health condition	Rotator cuff tendinopathy	Musculoskeletal	> 18	86	54.7	Balanced
Leung, Y.-Y., 2016	Case-control study (Intervention study)	China (Hong Kong)	_	Single health condition	Inflammatory arthritis	Musculoskeletal	> 18	99	51.9	Female predominance
Kono, A., 2016	Randomized controlled trial (Intervention study)	Japan	Urban	Fragility or functional decline	_	_	> 65	360	79.2	Female predominance

Kjerstad, E., 2016	Randomized controlled trial (Intervention study)	Norway	Rural	Fragility or functional decline	_	_	> 18	46	79	Female predominance
Jones, F., 2016	Randomized controlled trial (Intervention study)	United Kingdom	_	Single health condition	Stroke	Neurological	_	78	65.25	Balanced
Cameron-Tucker, H. L., 2016	Randomized controlled trial (Intervention study)	Australia	_	Single health condition	COPD	Respiratory	> 18	65	69	Balanced
Calugi, S., 2016	Quasi-experimental trial (Intervention study)	Italy	_	Single health condition	Stroke	Respiratory	> 18	229	70.95	Male predominance
Bleijenberg, N., 2016	Randomized controlled trial (Intervention study)	Netherlands	_	Fragility or functional decline	_	_	> 60	3092	74	Balanced
Van Dijk-de Vries, A., 2015	Randomized controlled trial (Intervention study)	Netherlands	_	Single health condition	Diabetes	Metabolic	_	264	64.5	Balanced
Van der Weegen, S., 2015	Randomized controlled trial (Intervention study)	Netherlands	_	Single health condition	COPD	Respiratory	< 70	199	57.8	Balanced
Scharlach, A. E., 2015	Cohort study (Observational Study)	United States of America	Urban	Fragility or functional decline	_	_	> 60	96	76	Female predominance
Pighills, A. C., 2015	Randomized controlled trial (Intervention study)	Australia	Urban	Fragility or functional decline	_	_	> 65	153	75.6	Balanced
Mosleh, S. M., 2015	Cohort study (Observational Study)	Scotland	_	Single health condition	Cardiovascular disease not specified	Cardiovascular	_	305	61.1	Male predominance
Mays, R. J., 2015	Pilot randomized controlled trial (Intervention study)	United States of America	_	Single health condition	Peripheral artery disease	Cardiovascular	Other	20	65.35	Female predominance
Lou, P., 2015	Randomized controlled trial (Intervention study)	China	Rural	Single health condition	COPD	Respiratory		8217	61.5	Balanced
Kidd, L., 2015	Mixed methods (Intervention study)	United Kingdom	_	Single health condition	Stroke	Neurological	_	26	64	Balanced
Garvey, J., 2015	Randomized controlled trial (Intervention study)	Ireland	_	Multimorbidity	_	_	> 18	50	66.25	Female predominance
Forster, A., 2015	Randomized controlled trial (Intervention study)	United Kingdom	_	Single health condition	Stroke	Neurological	_	800	71.7	Balanced
Foley, M. P., 2015	Pretest-posttest (Intervention study)	United States of America	_	Single health condition	Cancer not specified	Cancer	> 18	59	59	Female predominance
Clark, R. A., 2015	Mixed methods (Intervention study)	Australia	Rural	Single health condition	Heart failure	Cardiovascular	> 18	5	61.6	_
Martel, D., 2018	Randomized controlled trial (Intervention study)	Canada	_	Fragility or functional decline	_	_	> 65	44	73.5	Female predominance

Hevey, D., 2020	Pretest-posttest (Intervention study)	United Kingdom	Urban	Multimorbidity	_	_	_	273	_	Female predominance
King, A. I. I., 2018	Qualitative study (Descriptive study)	New Zealand	_	Fragility or functional decline	_	_	> 75	5	_	_
De Vriendt, P., 2016	Randomized controlled trial (Intervention study)	Belgium	_	Fragility or functional decline	_	_	> 65	168	80.4	Female predominance
Langoni, C. D. S., 2019	Randomized controlled trial (Intervention study)	Brazil	Urban	Fragility or functional decline	_	_	> 60	52	72.25	Female predominance
Winkel, A., 2015	Pretest-posttest (Intervention study)	Denmark	Rural	Fragility or functional decline	_	_	> 65	91	79.6	Female predominance
Lewin, G., 2016	Model development (Descriptive study)	Australia	_	Fragility or functional decline	_	_	_	58	76.8	Female predominance
Metzelthin, S. F., 2015	Randomized controlled trial (Intervention study)	Nethelerlands	_	Fragility or functional decline	_	_	> 70	346	77.2	
Liang, C. C., 2022	Observational Study (Observational Study)	Taiwan	_	Fragility or functional decline	_	_	> 65	56	76.5	Female predominance
Stathi, A., 2022	Pragmatic, multicentre, two-arm, single-blind, parallel-group, randomised, controlled trial (Intervention study)	UK	Urban,Rural	Fragility or functional decline	_	_	> 65	777	77.6	Female predominance
Tekin, F., 2022	Randomized-controlled trial (Intervention study)	Turkey	_	Fragility or functional decline	_	_	> 65	255	69	Balanced
Otero, P., 2021	Quasi-experimental study (Intervention study)	Spain	Urban	Fragility or functional decline	_	_	Other	25	54.9	Female predominance
Kim, S., 2021	Quasi-experimental study (Intervention study)	Korea	Rural	Fragility or functional decline	_	_	> 65	224	75	Female predominance
Sun, F. C., 2021	Quasi-experimental study (Intervention study)	Taiwan	_	Fragility or functional decline	_	_	> 65	122	73.9	Female predominance
Farinha, C., 2021	Randomized controlled trial (Intervention study)	Portugal	Rural	Fragility or functional decline	_	_	> 65	102	71.4	Female predominance
Barker, K. L., 2021	Randomized controlled trial (Intervention study)	England	_	Single health condition	Osteoarthritis	Musculoskeletal	Other	621	70.4	Balanced

Meisingset, I., 2021	Longitudinal observational study (Observational Study)	Norway	_	Fragility or functional decline	_	_	> 65	603	84	Female predominance
Suikkanen, S., 2021	Randomized controlled trial (Intervention study)	Finland	Urban,Rural	Fragility or functional decline	_	_	> 65	300	82.2	Female predominance
Yi, D., 2021	Experimental study (Intervention study)	Korea	_	Single health condition	COVID-19	Respiratory, Communicable diseases	> 65	70	76.7	Female predominance
Ullrich, P., 2022	Randomized Controlled Trial (Intervention study)	Germany	_	Multimorbidity	_	_	> 65	118	82.3	Female predominance
Hosteng, K. R., 2021	Intervention study (Intervention study)	United States of America	_	Fragility or functional decline	_	_	> 65	68	81.2	Female predominance
Sen, E. I., 2021	Randomized controlled trial (Intervention study)	Turkey	_	Single health condition	Sarcopenia	Musculoskeleta l	< 80	90	72.8	Female predominance
Sok, S., 2021	Quasi-experimental study (Intervention study)	Korea	_	Fragility or functional decline	_	_	> 65	65	73.7	Female predominance
Jungreitmayr, S., 2021	Intervention study	Austria,Italy	_	Fragility or functional decline	_	_	< 85	72	75.5	Female predominance
Liang, C. K., 2021	Randomized controlled trial (Intervention study)	Taiwan	Urban,Rural	Fragility or functional decline	_	_	> 65	733	74	Female predominance
Furuta, K., 2022	Pilot intervention study (Intervention study)	Japan	_	Fragility or functional decline	_	_	< 85	25	76	Female predominance
Oh, S. L., 2021	Intervention study (Intervention study)	South Korea	Rural	Single health condition	Osteoarthriti s	Musculoskeleta l	_	60	71.7	Female predominance
Tou, N. X., 2021	Randomized controlled trial (Intervention study)	Singapore	_	Fragility or functional decline	_	_	Other	57	71.7	Female predominance
Borges-Machado, F., 2021	Quasi-experimental study (Intervention study)	Portugal	Urban	Single health condition	Cognitive Impairment including dementia or aphasia	Neurological	> 60	43	78.2	Female predominance
Pepera, G., 2021	Randomized parallel-group study (Intervention study)	Greece	_	Fragility or functional decline	_	_	> 65	40	79.4	Female predominance
Arena, S. K., 2021	Randomized controlled trial (Intervention study)	United States of America	_	Fragility or functional decline	_	_	> 65	110	76.9	Female predominance

Mao, H. F., 2021	Pre-post intervention study (Intervention study)	Taiwan	_	Fragility or functional decline	_	_	> 65	130	78.2	Female predominance
Bagkur, M., 2021	Pre-post intervention study (Intervention study)	Cyprus	Rural	Fragility or functional decline	_	_	< 90	23	72.4	Female predominance
Woo, J., 2021	Narrative model description (Descriptive study)	China (Hong Kong)	_	Fragility or functional decline	_	_	_	102	84	Female predominance

(_)Information not provided or not applicable. * Total number of participants, including the sum of the intervention and control groups, if applicable. **Where the overall mean age of participants was reported by group only, we summed the reported age and divided by the number of groups. ***Male predominance= More than 60% of study participants were males. Female predominance= Less than 40% of study participants were males. Balanced= Males represent between 40% and 60% of study participants

Table S3. Rehabilitation programs' most important characteristics

First author, year	Name of the overall intervention	Mode of service delivery	Rehabilitation interventions	Co-design of intervention*	Providers	Health care workers	Multidisciplinary rehabilitation team**	Role or task shifthig***	Integrated care****
Kim, H., 2021	Systems for Person-centered Elder Care (SPEC)	Eldercare, Telerehabilitation	Assessment of frailty, Comprehensive assessment of functioning, Case management, Assessment of person-centered goals and priorities for health care, Caregivers education and training	_	Health workers	Nurse	No	No	Yes
Baba, Y., 2021	Adachi Rehabilitation program (ARP)	Eldercare	Therapeutic exercise, Occupational therapy	_	Health workers	Other	No	No	No
Miller, J., 2020	Chronic Pain Self-Management Support With Pain Science Education and Exercise (COMMENCE)	Community	Education and counseling in self-management, Behavioral interventions	_	Health workers	Physical therapist	No	No	No
Nøst, T. H., 2018	Groupbased chronic pain self-management course	Community	Education and counseling in self-management, Education and counseling on physical activity or exercise, Psychological interventions, Behavioral interventions	Yes	Health workers	Physical therapist	No	No	No
Nøst, T. H., 2018	Chronic pain self-management intervention	Community	Education and counseling in self-management, Psychological interventions, Behavioral interventions, Therapeutic exercise, Peer support group	Yes	Health workers	Physical therapist	No	No	No
Mendoza-Núñez, V. M., 2018	Healthy aging program workshop	Community	Education and counseling in self-management	_	Health workers	Geriatrician	No	No	No
Markle-Reid, M., 2018	Self-management program	Community, Home	Assessment of person-centered goals and priorities for health care, Education and counseling on nutrition, Education and counseling in self-management, Follow-up, Peer support or peer support group, Case	Yes	Health workers, Peers and volunteers	Nurse, Dieticians, Other	Yes	Yes	No

			management, Motivational activities, Home visits						
Lycholip, E., 2018	Information and communication technology (ICT)-guided disease management system (ICT-guided DMS)	Telerehabilitation	Assessment of health status, Health status monitoring Education and counseling in self-management	Yes	Health workers	Nurse	No	No	No
Lachance, L., 2018	Evidence-based chronic disease prevention and management interventions	Community	Peer support or peer support group, Education and counseling in self-management, Social care and support plan, Case management, Caregivers education and training	_	Health workers	Community health worker	No	No	No
Wong, A. K. C., 2019	Self-care promoting program	Community, Home	Comprehensive assessment of functioning ,Assessment of person-centered goals and priorities for health care, Education and counseling in self-management, Social care and support plan, Case management, Assessment of environment ,Follow-up	_	Health workers	Nurse, Community health worker, Social workers	Yes	No	No
Kamwesiga, J. T., 2018	F@ce™= a mobile phone supported family-centred ADL intervention	Telerehabilitation	Assessment of person-centered goals and priorities for health care, Occupational therapy, Activities of daily living skills training, Caregivers education and training	_	Health workers, Informal caregivers and family	Occupational Therapist	No	Yes	No
Von Storch, K., 2019	Telemedicine-assisted self-management program	TeleRehabilitation	Assessment of person-centered goals and priorities for health care, Health status monitoring, Education and counseling in self-management, Behavioral interventions, Motivational activities	_	_	_	_	No	No
Inzitari, M., 2018	Health Challenges program	Outpatient, Community	Assessment of frailty,Assessment of health status, Comprehensive assessment of functioning, Optimization of pharmacological therapy, Assessment of nutritional status, Therapeutic exercise,Education and counseling on physical activity or exercise, Education and counseling in self-management, Education and counseling on nutrition	Yes	Health workers	Geriatrician, Nurse, Physical therapist, Social workers, General practitioner	Yes	No	Yes
Godtfredsen, N., 2018	Standardized multidisciplinary pulmonary rehabilitation program	Community	Assessment of health status, Therapeutic exercise, Education and counseling in self-management, Education and counseling on nutrition, Social care and support plan, Psychological interventions, Education and counseling on physical activity or exercise	_	Health workers	Nurse, Physical therapist, General practitioner, Dieticians	Yes	No	Yes
Ehde, D. M., 2018	Telehealth Self-Management and Education Intervention	TeleRehabilitation, Community	Follow-up, Education and counseling in self-management, Behavioral interventions, Education and counseling on nutrition	_	Health workers	Therapist not specified	No	No	No

Nilsson B. B., 2019	Norwegian Ullevaal model	Outpatient	Therapeutic exercise, Education and counseling in self-management, Motivational activities	_	Health workers	Physical therapist, Other Physicians, Psychologist	Yes	No	No
Dye, C., 2018	Community Health Coaching	Home, Telerehabilitation	Education and counseling in self-management, Health status monitoring, Assessment of health status, Comprehensive assessment of functioning, Assessment of person-centered goals and priorities for health care, Assessment of fall risk, Follow-up, Education and counseling on nutrition, Education and counseling on physical activity or exercise, Social care and support plan, Case management, Caregivers education and training	Yes	Peers and volunteers	_	No	No	Yes
Dean, S. G., 2018	Rehabilitation Training (ReTrain) intervention	Community, Home	Assessment of person-centered goals and priorities for health care, Therapeutic exercise,Education and counseling in self-management, Education and counseling on physical activity or exercise, Follow-ups visits	_	Health workers	Community health worker	No	No	No
Clevenger, C. K., 2018	Integrated Memory Care Clinic	Eldercare	Assessment of person-centered goals and priorities for health care, Social care and support plan, Caregivers education and training	_	Health workers, Informal caregivers and family	Geriatrician, Nurse, Social workers	Yes	Yes	Yes
Zhang, L., 2017	Community health service center (CHSC)-based Cardiac Rehabilitation (CR)	Home, Community	Comprehensive assessment of functioning, Assessment of emotional functions, Follow-up, Therapeutic exercise ,Education and counseling on Healthy Lifestyle Behaviors, Peer support or peer support group, Caregivers education and training	_	Health workers	Physical therapist, Nurse, Dieticians, Psychologist, Other Physicians	Yes	No	No
Vega-Ramírez, F. A., 2017	Homebased rehabilitation (HBR) intervention	Home	Therapeutic exercise, Activities of daily living skills training, Assessment of person-centered goals and priorities for health care, Assessment of fall risk, Social care and support plan, Caregivers education and training	_	Health workers, Informal caregivers and family	PRM physicians, Physical therapist, Occupational Therapist	Yes	Yes	No
Uittenbroek, R., 2017	Embrace model	Community	Social care and support plan, Assessment of person-centered goals and priorities for health care, Follow-up, Health status monitoring, Functioning monitoring, Education and counseling in self-management, Rehabilitation coordination and management	_	Health workers	Geriatrician, Nurse, Social workers, Gereral parctitioner	Yes	No	Yes

Ru, X., 2017	Community-based rehabilitation appropriate technique (CRAT) intervention program	Community, Home	Comprehensive assessment of functioning, Multicomponent rehabilitation programme, Education and counseling in self-management, Caregivers education and training	_	Health workers	Community health worker	No	No	No
Whitehead, P. J., 2018	Bathing adaptations in the homes of older adults (BATH-OUT)	Home	Assessment of environment ,Environmental modifications	_	Health workers	Occupational Therapist	No	No	No
Van Lieshout, M. R. J., 2018	Proactive Multicomponent Intervention program	Community	Social skills training, Therapeutic exercise, Education and counseling on self-management, Problem-solving skills training, Cognitive training, Optimization of pharmacological therapy, Education and counseling on nutrition	_	Health workers	Physical therapist, Nurse, Dieticians	Yes	No	No
Valdivieso, B., 2018	Telephone-based, nurse-led case management program	Telerehabilitation	Health status monitoring, Follow-up, Education and counseling in self-management, Caregivers education and training, Social care and support plan	_	Health workers	Nurse	No	No	No
Taube, E., 2018	Case management intervention	Home	Assessment of health status, Comprehensive assessment of functioning, Assessment of person-centered goals and priorities for health care, Assessment of polypharmacy, Assessment of emotional functions, Assessment of fall risk, Social care and support plan ,Emotional support, Therapeutic exercise, Social skills training, Case management, Follow-up, Health status monitoring, Functioning monitoring	_	Health workers	Physical therapist, Nurse	No	No	No
Kitzman, P., 2017	The Kentucky Care Coordination for Community Transitions (KC3T) program	Community,Home	Case management, Discharge planning, Follow-up, Assessment of environment, Environmental modifications, Social care and support plan, Education and counseling in self-management, Caregivers education and training, Home visits	_	Health workers	Community health worker	No	No	Yes
Cecins, N., 2017	Community-based Pulmonary rehabilitation programs (PRPs)	Community	Therapeutic exercise, Pharmacological therapy, Education and counseling in self-management, Follow-up	_	Health workers	Physical therapist	No	No	No
Bleijenberg, N., 2017	Nurse-Led Care programs= U-PROFIT and HCP	Home	Case management, Follow-up, Functioning monitoring, Health status monitoring, Assessment of person-centered goals and priorities for health care, Comprehensive geriatric assessment, Assessment of family and ceregivers' needs, knowledge, and skills, Multidisciplinary geriatric care ,Education and counseling in self-management,	_	Health workers	Nurse	No	No	Yes

			Caregivers education and training, Management of urinary incontinence						
Barker, R. N., 2017	Community Rehabilitation northern Queensland (CRnQ) service	Outpatient	Assessment of person-centered goals and priorities for health care, Rehabilitation coordination and management, Multicomponent rehabilitation programme, Education and counseling in self-management, Follow-up	_	Health workers	Physical therapist, Dieticians, Speech and language therapists, Occupation al Therapist, Nurse, Social workers	Yes	No	No
Zakrisson, A.-B., 2016	Nurse-led multidisciplinary pulmonary rehabilitation (PR) program	Outpatient	Education and counseling in self-management, Therapeutic exercise, Education and counseling on Healthy Lifestyle Behaviors, Caregivers education and training	_	Health workers	Physical therapist, Occupation al Therapist, Nurse, Social workers, Dieticians	Yes	No	No
Young-Mee, K., 2016	Wholeness program for Brain Health	Community	Therapeutic exercise, Psychological interventions, Emotional support, Therapeutic recreation, Motivational activities	_	Health workers	Other, Social workers	No	No	No
Vorrink, S. N. W., 2016	mHealth intervention	Telerehabilitation	Assessment of person-centered goals and priorities for health care, Therapeutic exercise, Health status monitoring, Functioning monitoring	_	Health workers	Physical therapist	No	No	No
Tarazona-Santabalbina, F. J., 2016	Multicomponent exercise program (MEP)	Outpatient	Therapeutic exercise, Health status monitoring	_	Health workers	Physical therapist, Nurse	No	No	No
Shinkai, S, 2016	Health Literacy and Exercise programs	Community	Comprehensive geriatric assessment, Functioning monitoring, Education and counseling on Healthy Lifestyle Behaviors, Education and counseling on physical activity or exercise, Therapeutic exercise, Social care and support plan, Education and counseling on nutrition	Yes	Health workers	General practitione r, Nurse	No	No	No
Ruikes, F. G. H., 2016	CareWell primary care program	Outpatient	Assessment of polypharmacy, Rehabilitation coordination and management, Case management, Functioning monitoring, Follow-up,Health status monitoring, Assessment of person-centered goals and	_	Health workers	Geriatrician , Nurse, General practitione r	Yes	No	No

			priorities for health care, Social care and support plan						
Rosen, D., 2016	Tele-coaching intervention	Telerehabilitation	Case management, Education and counseling on nutrition, Functioning monitoring, Follow-up, Health status monitoring, Assessment of person-centered goals and priorities for health care, Education and counseling in self-management, Behavioral interventions, Education and counseling on Healthy Lifestyle Behaviors	_	Health workers	Social workers	No	No	No
McNamara, R. J., 2016	Community-based supervised exercise training program	Community	Therapeutic exercise	_	Health workers	Physical therapist	No	No	No
Mas, M. À., 2016	Hospital-at-home integrated care program	Home	Comprehensive geriatric assessment, Assessment of person-centered goals and priorities for health care, Rehabilitation coordination and management, Activities of daily living skills training, Therapeutic exercise, Environmental modifications, Assessment of fall risk	_	Health workers	Physical therapist, Occupational Therapist, Other Physicians, Nurse	Yes	No	Yes
Marsden, D. L., 2016	Home- and community based exercise program	Community	Comprehensive assessment of functioning, Therapeutic exercise, Assessment of person-centered goals and priorities for health care, Follow-up, Education and counseling on physical activity or exercise	_	Health workers	Physical therapist	No	No	No
Looman, W. M., 2016	Pro-active integrated care intervention	Outpatient	Multicomponent rehabilitation programme, Comprehensive assessment of functioning, Assessment of cognitive functions, Assessment of emotional functions, Assessment of person-centered goals and priorities for health care, Rehabilitation coordination and management, Case management, Assessment of fall risk, Assessment of polypharmacy, Management of urinary and bowel incontinence	_	Health workers	Physical therapist, General practitioner, Nurse, Other	Yes	No	Yes
Littlewood, C., 2016	Self-managed single exercise programme	Outpatient	Physical therapy, Follow-up, Education and counseling on physical activity or exercise	_	Health workers	Physical therapist	No	Yes	No
Leung, Y.-Y., 2016	Arthritis self-management program (ASMP)	Community	Education and counseling in self-management, Peer support or peer support group, Assessment of person-centered goals and priorities for health care, Therapeutic exercise	_	Health workers, Peers and volunteers	Nurse	No	Yes	No
Kono, A., 2016	Preventive Home Visit (PHV) program	Home	Comprehensive assessment of functioning, Functioning monitoring, Assessment of cognitive functions, Assessment of person-	_	Health workers	Nurse, Social	Yes	No	No

			centered goals and priorities for health care, Rehabilitation coordination and management			workers, Other			
Kjerstad, E., 2016	Reablement intervention	Home	Comprehensive assessment of functioning, Assessment of person-centered goals and priorities for health care, Caregivers education and training, Education and counseling in self-management, Activities of daily living skills training, Provision and training in the use of assitive products, Environmental modifications, Social care and support plan, Occupational therapy, Physical therapy	_	Health workers	Physical therapist, Occupation al Therapist	No	No	No
Jones, F., 2016	Self-management program	Community	Multicomponent rehabilitation programme, Assessment of person-centered goals and priorities for health care, Education and counseling in self-management	_	Health workers	Occupation al Therapist, Physical therapist, Speech and language therapists	Yes	No	Yes
Cameron-Tucker, H. L., 2016	Telephone-mentoring with home-based walking	Telerehabilitation	Assessment of person-centered goals and priorities for health care, Therapeutic exercise,Education and counseling on Healthy Lifestyle Behaviors, Education and counseling in self-management, Follow-up	_	Health workers	Nurse	No	No	No
Calugi, S., 2016	Adaptive physical activity (APA) program combined with therapeutic patient education (TPE)	Community	Caregivers education and training, Education and counseling in self-management, Education and counseling on Healthy Lifestyle Behaviors, Therapeutic exercise	_	Health workers	Other Physicians, Physical therapist	No	No	No
Bleijenberg, N., 2016	Proactive Primary Care Program	Outpatient	Assessment of frailty, Assessment of polypharmacy, Comprehensive geriatric assessment, Functioning monitoring, Case management, Follow-up, Multicomponent rehabilitation programme, Management of urinary incontinence	_	Health workers	General practitione r, Nurse	No	No	No
Van Dijk-de Vries, A., 2015	Self-Management Support (SMS)	Outpatient	Assessment of person-centered goals and priorities for health care, Comprehensive assessment of functioning,Assessment of emotional functions, Emotional support, Education and counseling in self-management, Problem solving skills training	_	Health workers	Nurse	No	Yes	No
Van der Weegen, S., 2015	It's LiFe! Mobile and Web-Based Monitoring and Feedback Tool	TeleRehabilitatio n	Education and counseling in self-management, Motivational activities, Education and counseling on physical activity or exercise, Functioning monitoring,	Yes	Health workers	Nurse	No	No	No

			Assessment of person-centered goals and priorities for health care						
Scharlach, A. E., 2015	ElderHelp Concierge Club: an integrated community-based care model	Home	Comprehensive assessment of functioning, Case management, Assessment of environment, Assistance in activities of daily living	_	Peers and volunteers	_	No	No	No
Pighills, A. C., 2015	Professional skill-sharing intervention	Community	Comprehensive assessment of functioning, Rehabilitation coordination and management, Multicomponent rehabilitation programme	_	Health workers	Physical therapist, Occupational Therapist, Other Physicians	Yes	Yes	No
Mosleh, S. M., 2015	Community-based cardiac rehabilitation	Outpatient	Therapeutic exercise, Education and counseling in self-management	_	Health workers	Physical therapist, Nurse, Psychologist, Dieticians	Yes	No	No
Mays, R. J., 2015	Walking exercise intervention	Community	Therapeutic exercise, Education and counseling in self-management, Assessment of environment, Functioning monitoring	_	_	_	_	No	No
Lou, P., 2015	Health management program	Community	Rehabilitation coordination and management, Comprehensive assessment of functioning, Follow-up, Education and counseling in self-management, Education and counseling on Healthy Lifestyle Behaviors	_	Health workers	General practitioner, Nurse, PRM physicians, Dieticians	Yes	No	No
Kidd, L., 2015	Self-management intervention	Home	Motivational activities, Assessment of person-centered goals and priorities for health care, Education and counseling in self-management	Yes	Health workers	Nurse	No	No	No
Garvey, J., 2015	OPTIMAL= occupation-based self-management programme	Community	Education and counseling in self-management, Assessment of person-centered goals and priorities for health care, Education and counseling on Healthy Lifestyle Behaviors, Peer support or peer support group	_	Health workers	Occupational Therapist	No	No	No
Forster, A., 2015	System of Longer-Term Stroke Care	Community	Comprehensive assessment of functioning, Multicomponent rehabilitation programme	_	Health workers	_	_	No	No
Foley, M. P., 2015	The Central Savannah River Area (CSRA) LIVESTRONG program	Community	Therapeutic exercise, Comprehensive assessment of functioning, Assessment of person-centered goals and priorities for health care	_	Health workers	Physical therapist	No	No	No
Clark, R. A., 2015	Tablet computer-based educational resource	TeleRehabilitation	Education and counseling in self-management	_	Health workers	_	_	No	No
Martel, D., 2018	Home-based (HEPtech) and community-based	Outpatient,TeleRehabilitation	Therapeutic exercise, Follow-up	_	Health workers	Physical therapist	No	No	No

	(YMCA) physical activity programs								
Hevey, D., 2020	Chronic Disease Self-Management Program (CDSMP)	Community	Peer support or peer support group, Education and counseling in self-management, Assessment of person-centered goals and priorities for health care, Behavioral interventions	_	Peers and volunteers	_	No	Yes	No
King, A. I. I., 2018	Implementation of a screening tool by a gerontology nurse	Community	Comprehensive assessment of functioning, Comprehensive geriatric assessment, Functioning monitoring, Assessment of polypharmacy, Case management, Follow-up	Yes	Health workers, Peers and volunteers	General practitioner, Nurse	No	Yes	No
De Vriendt, P., 2016	Client-Centred and Activity-Oriented Program	Community	Assessment of person-centered goals and priorities for health care, Activities of daily living skills training, Provision and training in the use of assitive products, Caregivers education and training	_	Health workers	Occupational Therapist	No	No	Yes
Langoni, C. D. S., 2019	Group exercises	Community	Therapeutic exercise, Motivational activities	_	_	_	_	No	No
Winkel, A., 2015	Reablement in a community setting	Home	Case management,Follow-up, Assessment of person-centered goals and priorities for health care, Activities of daily living skills training, Assessment of environment, Environmental modifications, Caregivers education and training	_	Health workers	Occupational Therapist	No	No	No
Lewin, G., 2016	The Home Independence program	Home	Comprehensive assessment of functioning, Therapeutic exercise, Education and counseling in self-management, Assessment of person-centered goals and priorities for health care, Activities of daily living skills training, Provision and training in the use of assitive products, Multicomponent rehabilitation programme, Rehabilitation coordination and management, Pharmacological therapy, Education and counseling on nutrition, Management of urinary and bowel incontinence	_	Informal caregivers and family	_	No	Yes	No
Metzelthin, S. F., 2015	Prevention of Care (PoC) approach	Home	Assessment of frailty, Multicomponent rehabilitation programme, Assessment of person-centered goals and priorities for health care, Rehabilitation coordination and management	_	Health workers	Physical therapist, Occupational Therapist, General practitioner, Nurse	Yes	No	No
Liang, C. C., 2022	Volunteer-Led Community Care Station program	Community	Social care and support, Education and advice on self care, Home visits, Therapeutic exercises, Therapeutic recreation	_	Peers and volunteers	_	No	Yes	No

Stathi, A., 2022	Physical activity and behaviour maintenance program	Community	Therapeutic exercise, Therapeutic recreation, Education and advice on self care, Education to influence lifestyle behaviours, Behavioural interventions	_	Health workers	Exercise professionals	No	No	No
Tekin, F., 2022	Telerehabilitative Home Exercise program	TeleRehabilitation	Therapeutic exercise, Assessment of risk for falls, Assessment of functioning	_	Health workers	Physical therapist	No	No	No
Otero, P., 2021	Videogame for the Promotion of Active Aging	TeleRehabilitation	Cognitive training, Pshychological interventions, Education to influence lifestyle behaviours, Therapeutic recreation	_	_	_	_	No	No
Kim, S., 2021	Physical Exercise program	Community	Therapeutic excercise	_	Health workers	Exercise professionals, Community health worker	No	No	No
Sun, F. C., 2021	Group Music Therapy with Physical Activities	Community	Therapeutic excercise, Music therapy	_	Health workers	Physical therapist, Music therapist	No	No	No
Farinha, C., 2021	Aquatic Exercise program	Community	Therapeutic excercise	_	Health workers	Exercise professionals	No	No	No
Barker, K. L., 2021	Community Rehabilitation after Knee Arthroplasty (CORKA) program	Home	Assessment of functioning ,Assessment of person-centered goals and priorities for health care, Therapeutic excecise, Provision and training in the use of assistive products	Yes	Health workers	Physical therapist, Physical Therapist Assistants	No	Yes	No
Meisingset, I., 2021	Three different working models of home-based rehabilitation	Home, Eldercare	Therapeutic excercise, Home visits, Follow up visits, Occupational Therapy, Assessment of functioning, Assessment of person-centered goals and priorities for health care, Social care and support	_	Health workers	Physical therapist, Occupational Therapist, Home care personnel	Yes	No	No
Suikkanen, S., 2021	Structured, periodical, progressive, and multicomponent physical exercise program	Home	Therapeutic excercise, Education about nutrition, Education about physical activity and exercise	_	Health workers	Physical therapist	No	No	Yes
Yi, D., 2021	Remote Home-Based Exercise Program	Home,Telerehabilitation	Therapeutic Excercise, Assessment of risk for falls, Environmental adaptations, Home visits, Follow up visits	_	Health workers	Physical therapist	No	No	No
Ullrich, P., 2022	Home-based physical training and activity promotion program	Home	Education and advice on self care, Education about physical activity and exercise, Motivational interventions, Therapeutic excersie, Training for activities of daily living,	_	Health workers	Exercise professionals	No	Yes	No

			Assessment of environment, Assessment of functioning, Home visits						
Hosteng, K. R., 2021	MapTrek Intervention	Eldercare institution	Education about physical activity and exercise, Motivational interventions, Functioning monitoring, Education to influence lifestyle behaviours	Yes	_	_	_	_	No
Sen, E. I., 2021	The multicomponent exercise program (MCEP)	Home	Therapeutic excercise	_	_	_	_	_	No
Sok, S., 2021	Cognitive/exercise dual-task program	Community	Motivational interventions, Therapeutic exercise, cognitive training	_	Health workers	Nurse	No	Yes	No
Jungreitmayr, S., 2021	ICT-Supported Functional Fitness program (FFP)	Telerehabilitation	Therapeutic exercise	_	_	_	_	No	No
Liang, C. K., 2021	Multidomain intervention	Community	Therapeutic exercise, Education about nutrition, Cognitive training,Education and advice on self care	_	_	_	_	No	No
Furuta, K., 2022	Group program focused on enabling life performance	Community	Education and advice on self care, Assessment of person-centered goals and priorities for health care, Therapeutic exercise,Education about physical activity and exercise, Education to influence lifestyle behaviours	_	Health workers	Occupation al Therapist, Gerontolog ists	No	No	No
Oh, S. L., 2021	Community-based integrated exercise and health education program	Home,Outpatient	Therapeutic excercises, Education and advice on self care,Education to influence lifestyle behaviours, Education about nutrition, Counselling for weight management	_	Health workers	Exercise professiona ls, Physicians not specified , Dieticians, Nurse	Yes	No	No
Tou, N. X., 2021	Community-Delivered Functional Power Training program	Community	Therapeutic excercise	_	Health workers	Exercise professiona ls	No	No	No
Borges-Machado, F., 2021	Multicomponent intervention	Outpatient	Therapeutic excercise	_	Health workers	Exercise professiona ls	No	No	No
Pepera, G., 2021	Multicomponent exercise training intervention	Eldercare	Therapeutic excercise	_	Health workers	Physical therapist	No	No	No
Arena, S. K., 2021	HOP-UP-PT program	Community,teler ehabilitation	Therapeutic excercise, Assessment of risk for falls, Motivational interventions, Education to influence lifestyle behaviours, Environmental adapatations, Assessment of environment, Functioning monitoring	_	Health workers	Physical therapist	No	No	No
Mao, H. F., 2021	Multicomponent cognitive intervention	Community	Therapeutic excercise, Coginitve training, Training in activities of daily living,	_	Health workers	Occupation al Therapist	No	No	No

			Motivational interventions, Therapeutic recreation, Assessment of person-centered goals and priorities for health care						
Bagkur, M., 2021	Home-based interactive telerehabilitation program	Telerehabilitation	Therapeutic excercise	_	Health workers	Physical therapist	No	No	No
Woo, J., 2021	Integrated Model of Community Care	Outpatient,community	Therapeutic excercise, Assessment of risk for falls, Training for activities of daily living, Assessment of person-centered goals and priorities for health care, Cognitive training, Social care and support plan	_	Health workers	Nurse, Physical therapist, Dieticians, Optometrist	Yes	No	Yes

*Patients and/or caregivers were involved in the design of the intervention. This took place before the intervention and took into account patients' and/or caregivers' opinions, preferences and values. ***At least 3 providers were reported. ***The paper was consistent with the following definition of role or task shifting = a process by which services typically provided by one type of health worker are shifted to others with less extensive qualifications or training.****The paper was in line with the following definition of integrated care: Also known as integrated health, coordinated care, comprehensive care, seamless care, or transmural care, is a worldwide trend in health care reforms and new organizational arrangements focusing on more coordinated and integrated forms of care provision. OR the WHO definition, "Integrated care is a concept bringing together inputs, delivery, management and organization of services related to diagnosis, treatment, care, rehabilitation and health promotion. Integration is a means to improve services in relation to access, quality, user satisfaction and efficiency(1).

References:

1. Gröne O, Garcia-Barbero M. Integrated care: a position paper of the WHO European Office for Integrated Health Care Services. International journal of integrated care. 2001;1.
