# Supplementary material for: Rehabilitation in primary care for an ageing population: a secondary analysis from a scoping review of rehabilitation delivery models
Source: BMC Health Serv Res. 2024 Jan 23;24:123. doi: 10.1186/s12913-023-10387-w (PMC10804573; doi:10.1186/s12913-023-10387-w)
Supplement: Supplementary file 6 — Additional file 6. Rehabilitation dosage. [file 12913_2023_10387_MOESM6_ESM.pdf]

# Additional file 6

Table S4. Rehabilitation dosage

| First author, year | How was the intensity of rehabilitation decided? | Average of sessions' duration (in minutes) | Time of the longest session (in minutes) | Average of sessions' frequency number (per week) | Frequency of the most frequent session (per week) | Total number of sessions that each patient received | Total duration of the intervention (in weeks) |
|---|---|---|---|---|---|---|---|
| Kim, H., 2021 | Prespecified program, adapted considering patients' needs | _ | _ | 0.3 | _ | _ | 24 |
| Baba, Y., 2021 | Prespecified program | 105 | 150 | 1.0 | 1 | 4 | 4 |
| Miller, J., 2020 | Prespecified program, adapted considering patients' needs | 67.5 | 90 | 2.0 | 2 | 12 | 6 |
| Nøst, T. H., 2018 | Prespecified program | 150 | 150 | 1.0 | 1 | 6 | 6 |
| Nøst, T. H., 2018 | Prespecified program | 150 | 150 | 1.0 | 1 | 6 | 6 |
| Mendoza-Núñez, V. M., 2018 | Prespecified program | 300 | 300 | 1.0 | 1 | 20 | 20 |
| Markle-Reid, M., 2018 | Adapted considering patients' needs | _ | _ | 0.2 | _ | 15 | 24 |
| Lycholip, E., 2018 | Prespecified program, adapted considering patients' needs | _ | _ | _ | _ | _ | 36 |
| Lachance, L., 2018 | Prespecified program, adapted considering patients' needs | 100 | 150 | 1.8 | 3 | 54 | 10 |
| Wong, A. K. C., 2019 | Prespecified program | 25.6 | 60 | 0.1 | 0.1 | 8 | 12 |
| Kamwesiga, J. T., 2018 | Prespecified program, adapted considering patients' needs | _ | _ | 8.0 | 14 | 128 | 8 |
| Von Storch, K., 2019 | Prespecified program, adapted considering patients' needs | _ | _ | _ | _ | _ | 48 |
| Inzitari, M., 2018 | Prespecified program, adapted considering patients' needs | 60 | 60 | 1.0 | 1 | 10 | 10 |
| Godtfredsen, N., 2018 | Prespecified program, adapted considering patients' needs | _ | _ | 1.0 | 1 | 24 | 12 |
| Ehde, D. M., 2018 | Prespecified program | _ | _ | 1.0 | 1 | 8 | 8 |
| Nilsson B. B., 2019 | Prespecified program | 65 | 65 | 2.0 | 2 | 4 | 2 |
| Dye, C., 2018 | Prespecified program | 165 | 210 | 0.3 | 0.3 | 18 | 16 |
| Dean, S. G., 2018 | Prespecified program | 120 | 120 | 0.6 | 2 | 29 | 12 |
| Clevenger, C. K., 2018 | Adapted considering patients' needs | _ | _ | _ | _ | _ | 48 |
| Zhang, L., 2017 | Prespecified program, adapted considering patients' needs | 90 | 90 | 3.2 | _ | 120 | 12 |
| Vega-Ramírez, F. A., 2017 | Adapted considering patients' needs | _ | _ | 5.0 | 5 | 15 | 3 |
| Uittenbroek, R., 2017 | Adapted considering patients' needs | _ | _ | 0.5 | 0.5 | 24 | 48 |
| Ru, X., 2017 | Prespecified program | 90 | 90 | 2.0 | 2 | 24 | 12 |
| Whitehead, P. J., 2018 | Prespecified program, adapted considering patients' needs | _ | _ | _ | _ | 1 | 24 |

| | | | | | | | |
|---|---|---|---|---|---|---|---|
| Van Lieshout, M. R. J., 2018 | Prespecified program | 101.25 | 150 | 1.0 | 2 | 33 | 23 |
| Valdivieso, B., 2018 | Prespecified program | _ | _ | 0.3 | 0.6 | 32 | 48 |
| Taube, E., 2018 | Prespecified program, adapted considering patients' needs | _ | _ | 0.3 | 0.3 | 12 | 48 |
| Kitzman, P., 2017 | Prespecified program, adapted considering patients' needs | _ | _ | 0.8 | 0.8 | 18 | 24 |
| Cecins, N., 2017 | Prespecified program, adapted considering patients' needs | _ | _ | 2.0 | 2 | 16 | 8 |
| Bleijenberg, N., 2017 | Prespecified program | 34.3 | 34.3 | 0.1 | 0.1 | 7 | 36 |
| Barker, R. N., 2017 | Adapted considering patients' needs | _ | _ | 1.5 | 1.5 | 36 | 12 |
| Zakrisson, A.-B., 2016 | Prespecified program | 120 | 120 | 1.0 | 1 | 6 | 6 |
| Young-Mee, K., 2016 | Prespecified program | 90 | 90 | 1.0 | 1 | 12 | 12 |
| Vorrink, S. N. W., 2016 | Prespecified program, adapted considering patients' needs | _ | _ | _ | _ | _ | 48 |
| Tarazona-Santabalbina, F. J., 2016 | Prespecified program | 65 | 65 | 5.0 | 5 | 120 | 24 |
| Shinkai, S, 2016 | Prespecified program | 90 | 90 | 0.5 | 0.5 | 8 | 16 |
| Ruikes, F. G. H., 2016 | Prespecified program | _ | _ | 0.1 | 0.1 | 6 | 48 |
| Rosen, D., 2016 | Prespecified program | _ | _ | 1.0 | 1 | 4 | 4 |
| McNamara, R. J., 2016 | Adapted considering patients' needs | _ | _ | 2.0 | 2 | 16 | 8 |
| Mas, M. À., 2016 | Prespecified program, adapted considering patients' needs | _ | _ | _ | _ | _ | 144 |
| Marsden, D. L., 2016 | Prespecified program, adapted considering patients' needs | _ | _ | 2.0 | 2 | 16 | 8 |
| Looman, W. M., 2016 | Adapted considering patients' needs | _ | _ | _ | _ | _ | 48 |
| Littlewood, C., 2016 | Prespecified program | _ | _ | 14.0 | 14 | 672 | 48 |
| Leung, Y.-Y., 2016 | Prespecified program | 120 | 120 | 1.0 | 1 | 6 | 6 |
| Kono, A., 2016 | Adapted considering patients' needs | _ | _ | 0.1 | 0.1 | 8 | 96 |
| Kjerstad, E., 2016 | Adapted considering patients' needs | _ | _ | _ | _ | _ | 10 |
| Jones, F., 2016 | Adapted considering patients' needs | _ | _ | _ | _ | _ | 12 |
| Cameron-Tucker, H. L., 2016 | Prespecified program, adapted considering patients' needs | _ | _ | 2.0 | 2 | 24 | 12 |
| Calugi, S., 2016 | Prespecified program | _ | _ | 1.2 | 2 | 19 | 8 |
| Bleijenberg, N., 2016 | Prespecified program, adapted considering patients' needs | _ | _ | _ | _ | _ | 6 |
| Van Dijk-de Vries, A., 2015 | Prespecified program | _ | _ | _ | _ | _ | 12 |
| Van der Weegen, S., 2015 | Prespecified program | _ | _ | 0.2 | 0.2 | 4 | 24 |
| Scharlach, A. E., 2015 | Adapted considering patients' needs | _ | _ | _ | _ | _ | 24 |
| Pighills, A. C., 2015 | Adapted considering patients' needs | _ | _ | _ | _ | _ | 16 |
| Mosleh, S. M., 2015 | Prespecified program | _ | _ | 1.5 | 2 | 24 | 8 |
| Mays, R. J., 2015 | Prespecified program, adapted considering patients' needs | 50 | 50 | 3.0 | 3 | 42 | 14 |

| | | | | | | | |
|---|---|---|---|---|---|---|---|
| Lou, P., 2015 | Prespecified program, adapted considering patients' needs | 60 | 60 | 0.4 | 0.5 | _ | 192 |
| Kidd, L., 2015 | Prespecified program | _ | _ | 1.0 | 1 | 4 | 4 |
| Garvey, J., 2015 | Prespecified program | 180 | 180 | 1.0 | 1 | 6 | 6 |
| Forster, A., 2015 | Adapted considering patients' needs | _ | _ | _ | _ | _ | 12 |
| Foley, M. P., 2015 | Prespecified program | 90 | 90 | 2.0 | 2 | 24 | 12 |
| Clark, R. A., 2015 | Prespecified program | _ | _ | _ | _ | _ | _ |
| Martel, D., 2018 | Prespecified program | 55 | 55 | 2.0 | 2 | 24 | 12 |
| Hevey, D., 2020 | Prespecified program | 150 | 150 | 1.0 | 1 | 6 | 6 |
| King, A. I. I., 2018 | Prespecified program, adapted considering patients' needs | _ | _ | _ | _ | _ | 48 |
| De Vriendt, P., 2016 | Adapted considering patients' needs | _ | _ | _ | _ | _ | 10 |
| Langoni, C. D. S., 2019 | Prespecified program, adapted considering patients' needs | 60 | 60 | 2.0 | 2 | 48 | 24 |
| Winkel, A., 2015 | Prespecified program, adapted considering patients' needs | 52.5 | 60 | 0.2 | 0.25 | 4 | 12 |
| Lewin, G., 2016 | Prespecified program, adapted considering patients' needs | _ | _ | _ | _ | _ | 48 |
| Metzelthin, S. F., 2015 | Prespecified program, adapted considering patients' needs | _ | _ | _ | _ | _ | 96 |
| Liang, C. C., 2022 | Prespecified program | 210 | 210 | 2.0 | 2 | 96 | 48 |
| Stathi, A., 2022 | Prespecified program | 41.7 | 60 | 1.0 | 1.2 | 149 | 52 |
| Tekin, F., 2022 | Prespecified program | _ | _ | 5.0 | 5 | 20 | 4 |
| Otero, P., 2021 | Prespecified program | 70 | 70 | 1.0 | 1 | 8 | 8 |
| Kim, S., 2021 | Prespecified program | 55 | 60 | 1.0 | 1 | 56 | 28 |
| Sun, F. C., 2021 | Prespecified program | 120 | 120 | 1.0 | 1 | 12 | 12 |
| Farinha, C., 2021 | Prespecified program | 45 | 45 | 2.0 | 2 | 56 | 28 |
| Barker, K. L., 2021 | Adapted considering patients' needs | _ | _ | _ | _ | _ | _ |
| Meisingset, I., 2021 | Adapted considering patients' needs | _ | _ | _ | _ | _ | _ |
| Suikkanen, S., 2021 | Prespecified program, adapted considering patients' needs | 60 | 60 | 2.0 | 2 | 16 | 8 |
| Yi, D., 2021 | Prespecified program | 40 | 40 | 2.0 | 2 | 16 | 8 |
| Ullrich, P., 2022 | Prespecified program | _ | _ | 0.4 | 0.4 | 5 | 12 |
| Hosteng, K. R., 2021 | Prespecified program | _ | _ | _ | _ | _ | 8 |
| Sen, E. I., 2021 | Prespecified program | 60 | 60 | 3.0 | 3 | 36 | 12 |
| Sok, S., 2021 | Prespecified program | 50 | 50 | 2.0 | 2 | 20 | 10 |
| Jungreitmayr, S., 2021 | Prespecified program | 10 | 10 | 2.0 | 2 | 64 | 32 |
| Liang, C. K., 2021 | Prespecified program, adapted considering patients' needs | 40 | 60 | 1.3 | 1.3 | 39 | 48 |
| Furuta, K., 2022 | Prespecified program | 90 | 90 | 0.5 | 0.5 | 16 | 16 |
| Oh, S. L., 2021 | Prespecified program | 50 | 50 | 0.3 | 0.3 | 10 | 20 |
| Tou, N. X., 2021 | Prespecified program | 60 | 60 | 2.0 | 2 | 24 | 12 |
| Borges-Machado, F., 2021 | Prespecified program | 60 | 60 | 2.0 | 2 | 48 | 24 |
| Pepera, G., 2021 | Prespecified program | 50 | 50 | 2.0 | 2 | 16 | 8 |

| | | | | | | | |
|---|---|---|---|---|---|---|---|
| Arena, S. K., 2021 | Prespecified program, adapted considering patients' needs | _ | _ | 0.2 | 0.3 | 22 | 28 |
| Mao, H. F., 2021 | Prespecified program | 120 | 120 | 1.0 | 1 | 12 | 12 |
| Bagkur, M., 2021 | Prespecified program | 40 | 40 | 3.0 | 3 | 24 | 8 |
| Woo, J., 2021 | Prespecified program, adapted considering patients' needs | _ | _ | _ | _ | _ | 12 |
